# Effect of a Low-Fat Vegan Diet on Body Weight, Insulin Sensitivity, Postprandial Metabolism, and Intramyocellular and Hepatocellular Lipid Levels in Overweight Adults: A Randomized Clinical Trial

**DOI:** 10.1001/jamanetworkopen.2020.25454

**Published:** 2020-11-30

**Authors:** Hana Kahleova, Kitt Falk Petersen, Gerald I. Shulman, Jihad Alwarith, Emilie Rembert, Andrea Tura, Martin Hill, Richard Holubkov, Neal D. Barnard

**Affiliations:** 1 Physicians Committee for Responsible Medicine, Washington, DC; 2 Department of Internal Medicine, Yale School of Medicine, New Haven, Connecticut; 3 Department of Cellular & Molecular Physiology, Yale School of Medicine, New Haven, Connecticut; 4 Metabolic Unit, CNR Institute of Neuroscience, Padua, Italy; 5 Institute of Endocrinology, Prague, Czech Republic; 6 School of Medicine, University of Utah, Salt Lake City; 7 George Washington University School of Medicine and Health Sciences, Washington, DC

## Abstract

**Importance:**

Excess body weight and insulin resistance lead to type 2 diabetes and other major health problems. There is an urgent need for dietary interventions to address these conditions.

**Objective:**

To measure the effects of a low-fat vegan diet on body weight, insulin resistance, postprandial metabolism, and intramyocellular and hepatocellular lipid levels in overweight adults.

**Design, Setting, and Participants:**

This 16-week randomized clinical trial was conducted between January 2017 and February 2019 in Washington, DC. Of 3115 people who responded to flyers in medical offices and newspaper and radio advertisements, 244 met the participation criteria (age 25 to 75 years; body mass index of 28 to 40) after having been screened by telephone.

**Interventions:**

Participants were randomized in a 1:1 ratio. The intervention group (n = 122) was asked to follow a low-fat vegan diet and the control group (n = 122) to make no diet changes for 16 weeks.

**Main Outcomes and Measures:**

At weeks 0 and 16, body weight was assessed using a calibrated scale. Body composition and visceral fat were measured by dual x-ray absorptiometry. Insulin resistance was assessed with the homeostasis model assessment index and the predicted insulin sensitivity index (PREDIM). Thermic effect of food was measured by indirect calorimetry over 3 hours after a standard liquid breakfast (720 kcal). In a subset of participants (n = 44), hepatocellular and intramyocellular lipids were quantified by proton magnetic resonance spectroscopy. Repeated measure analysis of variance was used for statistical analysis.

**Results:**

Among the 244 participants in the study, 211 (87%) were female, 117 (48%) were White, and the mean (SD) age was 54.4 (11.6) years. Over the 16 weeks, body weight decreased in the intervention group by 5.9 kg (95% CI, 5.0-6.7 kg; *P* < .001). Thermic effect of food increased in the intervention group by 14.1% (95% CI, 6.5-20.4; *P* < .001). The homeostasis model assessment index decreased (−1.3; 95% CI, −2.2 to −0.3; *P* < .001) and PREDIM increased (0.9; 95% CI, 0.5-1.2; *P* < .001) in the intervention group. Hepatocellular lipid levels decreased in the intervention group by 34.4%, from a mean (SD) of 3.2% (2.9%) to 2.4% (2.2%) (*P* = .002), and intramyocellular lipid levels decreased by 10.4%, from a mean (SD) of 1.6 (1.1) to 1.5 (1.0) (*P* = .03). None of these variables changed significantly in the control group over the 16 weeks. The change in PREDIM correlated negatively with the change in body weight (*r* = −0.43; *P* < .001). Changes in hepatocellular and intramyocellular lipid levels correlated with changes in insulin resistance (both *r* = 0.51; *P* = .01).

**Conclusions and Relevance:**

A low-fat plant-based dietary intervention reduces body weight by reducing energy intake and increasing postprandial metabolism. The changes are associated with reductions in hepatocellular and intramyocellular fat and increased insulin sensitivity.

**Trial Registration:**

ClinicalTrials.gov Identifier: NCT02939638

## Key Points

**Question:**

What are the effects of a low-fat vegan diet on body weight, insulin resistance, postprandial metabolism, and intramyocellular and hepatocellular lipid levels in overweight adults?

**Findings:**

In this 16-week randomized clinical trial, a low-fat plant-based dietary intervention reduced body weight by reducing energy intake and increasing postprandial metabolism, which was associated with reductions in hepatocellular and intramyocellular fat and increased insulin sensitivity.

**Meaning:**

A low-fat plant-based diet is an effective tool for reducing body weight and increasing insulin sensitivity and postprandial metabolism.

## Teaser

This randomized clinical trial assesses the effects of a 16-week low-fat vegan dietary intervention on body weight, insulin resistance, postprandial metabolism, and intramyocellular and hepatocellular lipid levels in overweight adults.

## Introduction

Overweight and associated diseases, particularly type 2 diabetes and metabolic syndrome, remain worldwide challenges. There is an urgent need for dietary interventions to address these problems and for a better understanding of how different dietary interventions work.

Obesity is uncommon in individuals whose diets are based on plant-derived foods.^1,2^ In clinical trials, such diets caused weight loss, for which 2 explanations have been offered.^3^ First, a high-fiber, low-fat diet has a low energy density, which reduces energy intake. Second, a low-fat, vegan diet increases the thermic effect of food, which accounts for approximately 10% of total energy expenditure.^4^ However, in the latter randomized clinical trial, the control group was following an active diet based on National Cholesterol Education Program guidelines.^5^ Because there was no untreated control group, the effect of a low-fat vegan diet on thermogenesis remains unclear.

Studies have reported that people following a vegan diet have lower concentrations of intramyocellular lipids compared with those following omnivorous diets, suggesting that by reducing intramyocellular or hepatocellular lipid levels, a plant-based diet may lead to increased mitochondrial activity and postprandial metabolism.^6,7^ This is particularly important because the accumulation of lipids in muscle and liver cells may also be associated with insulin resistance and type 2 diabetes.^8,9,10^ The aim of this study was to measure the effects of a low-fat vegan diet on body weight, insulin resistance, postprandial metabolism, and intramyocellular and hepatocellular lipid levels in overweight adults.

## Methods

### Study Design and Eligibility

This randomized clinical trial using a single-center, open parallel design was conducted between January 2017 and February 2019 in Washington, DC, in 4 replications (the trial protocol is given in Supplement 1). Adults aged 25 to 75 years with a body mass index (BMI) (calculated as weight in kilograms divided by height in meters squared) of 28 to 40 were enrolled. Exclusion criteria were diabetes, smoking, alcohol or drug use, pregnancy or lactation, and current use of a vegan diet. The additional exclusion criteria for the subset of participants undergoing the proton magnetic resonance spectroscopy were the presence of any metal implant, claustrophobia, BMI higher than 38, and waist circumference of more than 102 cm. The study protocol was approved by the Chesapeake Institutional Review Board. All participants gave written informed consent. This study followed the Consolidated Standards of Reporting Trials (CONSORT) reporting guideline.^11^

### Randomization and Study Groups

With use of a computer-generated system, participants were randomly assigned (in a 1:1 ratio) to an intervention group, which was asked to follow a low-fat vegan diet, or a control group, which was asked to make no diet changes. The randomization protocol could not be accessed by the participants or the staff allocating the participants into groups beforehand. Because assignment was done simultaneously, allocation concealment was unnecessary. The participants were not blinded to their group assignment.

The intervention diet (approximately 75% of energy from carbohydrates, 15% protein, and 10% fat) consisted of vegetables, grains, legumes, and fruits without animal products or added fats. Vitamin B_12_ was supplemented (500 μg/d). The intervention group attended weekly classes for detailed instruction and cooking demonstrations and received printed materials and small food samples. No meals were provided.

For both groups, alcoholic beverages were limited to 1 per day for women and 2 per day for men. All participants were asked to maintain their customary exercise habits and medications unless modified by their personal physicians.

### Outcomes

All measurements were performed at baseline and 16 weeks. The outcome assessors (K.F.P., G.I.S., and A.T.) were blinded to group assignment. The primary outcomes were body weight, insulin resistance, postprandial metabolism, and the concentrations of intramyocellular and hepatocellular lipids.

At baseline and at 16 weeks, dietary intake data over 3 consecutive days were collected and analyzed by staff members certified in the Nutrition Data System for Research, version 2016, developed by the Nutrition Coordinating Center, University of Minnesota, Minneapolis.^12^ In addition, study dietitians made unannounced telephone calls to assess participants’ dietary adherence. All study participants were asked not to alter their exercise habits and to continue their preexisting medication regimens for the duration of the study. Physical activity was assessed by the International Physical Activity Questionnaire.^13^

Laboratory assessments were made after an overnight fast. Height (baseline only) and weight were measured using a stadiometer and a calibrated digital scale, respectively. Body composition and visceral fat volume were assessed using dual energy x-ray absorptiometry (iDXA; GE Healthcare), which has been validated against computed tomography^14^ and magnetic resonance imaging.^15^ The measurement of total body fat and visceral fat had a coefficient of variation (CV) of 1.0% and 5.4%, respectively.^16,17^

Insulin secretion was assessed after a standardized liquid breakfast (Boost Plus, Nestle) (720 kcal, 34% of energy from fat, 16% protein, and 50% carbohydrate). Plasma glucose, immunoreactive insulin, and C-peptide concentrations were measured at 0, 30, 60, 120, and 180 minutes. Plasma glucose concentration was analyzed using the Hexokinase UV end point method (the intra-assay CV was 1.4%, and the inter-assay CV was 1.9%), and immunoreactive insulin and C-peptide concentrations were determined using insulin and C-peptide electro-chemiluminescence immunoassay (the intra-assay CVs were 5.1% and 3.8%, respectively, and the inter-assay CVs were 5.7% and 3.9%, respectively). Glycated hemoglobin level was measured by turbidimetric inhibition immunoassay (the intra-assay CV was 3.7%, and the inter-assay CV was 3.5%), and lipid concentrations were measured by enzymatic colorimetric methods (intra-assay CV: total cholesterol, 2.1%; high-density lipoprotein cholesterol, 2.4%; low-density lipoprotein cholesterol, 2.0%; and triglycerides 2.2%; inter-assay CV: total cholesterol, 2.7%; high-density lipoprotein cholesterol, 3.8%; low-density lipoprotein cholesterol, 3.0%; and triglycerides 3.2%). All test kits were made by Roche.

Insulin resistance was calculated using the homeostasis model assessment index.^18^ The predicted insulin sensitivity index (PREDIM) provided a validated measure of dynamic insulin sensitivity.^19^ Resting energy expenditure and postprandial metabolism were measured by indirect calorimetry (Cosmed Quark CPET) using a ventilated hood system (accuracy of measurement with a CV<1% and repeatability of measurement with a CV of 1.2%).^20,21^ Each measurement was performed for 15 minutes after an overnight fast and 30, 60, 120, and 180 minutes after the standard breakfast.

In a subset of 44 participants (23 in the intervention group and 21 in the control group), proton magnetic resonance spectroscopy was performed at the Magnetic Resonance Research Center, Yale School of Medicine. Hepatocellular and intramyocellular lipids were quantified by proton magnetic resonance spectroscopy at 4T (Bruker).^22^ This method has been shown to provide a precise quantification of fat fractions, with a mean bias of −1.1.% to 0.5%.^23^ Hepatocellular lipid content was measured by ^1^H respiratory-gated stimulated echo acquisition mode spectroscopy in a 15 × 15 × 15-mm^3^ voxel. Acquisition was synchronized to the respiratory cycle and triggered at the end of expiration. A water-suppressed lipid spectrum and a lipid-suppressed water spectrum were acquired in 3 locations of the liver to account for liver inhomogeneity, and the total lipid content was averaged and calculated. In addition, hepatocellular lipid content was corrected for transverse relaxation using the transverse relaxation times of 22 ms for water and 44 ms for lipid.^24^ Intramyocellular lipid content was measured in the soleus muscle using an 8.5-cm diameter circular ^13^C surface coil with twin, orthogonal circular 13-cm ^1^H quadrature coils. Scout images of the lower leg were obtained to ensure correct positioning of the participant and to define an adequate volume for localized shimming using the FastMap procedure.^25^

### Power Analysis

Sample size was based on the change in body weight, insulin resistance, and postprandial metabolism previously observed with a plant-based diet,^4^ with an α level of 0.05. The assumed change for body weight was a mean (SD) of 5.8 (3.2) kg in the intervention arm and 1 (3.2) kg in the control arm; for insulin sensitivity, the assumed change was 1.1 (2.1) in the intervention arm and 0.1 (2.1) in the control arm; and for the thermic effect of food, the assumed change was 4.7 (12) (area under the curve) in the intervention arm and 0.3 (9.4) in the control arm. For the primary efficacy comparison, a total of 22 participants (11 per arm) were required for 90% power to detect a significant treatment effect on body weight between the 2 study arms. For insulin sensitivity, a total of 142 participants (71 in each arm) were required for 90% power. Assuming that the treatment effect for postprandial metabolism was of the same magnitude at each of the 5 evaluation points used in metabolic assessment and that the SD was approximately 10.85 points for all observations, with 5 observations per participant correlated at a magnitude of 0.7 with each other, and assuming an attrition of 10%, the required sample size was 81 per group (162 total) for 80% power and 108 per group (216 total) for 90% power.

For the substudy assessing the role of intramyocellular and hepatocellular lipids in insulin sensitivity, a study^26^ from 2012 provided a basis for a power analysis. In that study, 7 lean individuals with insulin resistance followed a hypocaloric (1200 kcal/d) diet for 9 weeks. The mean (SD) intramyocellular lipid level decreased from 1.1% (0.2%) to 0.8% (0.1%). Assuming a mean (SD) change in the intramyocellular lipid level of 0.3% (0.2%) in the intervention arm and a mean change of 0 with a similar SD in the control arm, to have 90% power to detect a difference of this magnitude between, the 2 arms would each require 11 individuals (22 total). Because this was an exploratory substudy and variability in response to the diet was largely unknown, 20 participants were recruited per arm (a total of 40 participants).

### Statistical Analysis

For baseline characteristics, between-group *t* tests were performed for continuous variables and *χ^2^* or Fisher exact test for categorical variables. A repeated measure analysis of variance (ANOVA) model was used with between-person and within-person factors and interactions, including group, person, and time. The interaction between group and time was calculated for each variable. For thermic effect of food, minutes were included in the ANOVA model. Data from only individuals with measurements at both time points were included in the ANOVA model. Within each group, paired comparison *t* tests were calculated to test whether the changes from baseline to 16 weeks were statistically significant.

To eliminate skewed data distribution and heteroscedasticity, data were transformed to a gaussian distribution before further processing by a power transformation using the statistical software Statgraphics Centurion, version XV (Statpoint Inc). The transformed data underwent multivariable regression using the method of orthogonal projections to latent structure.^27^ This method is effective in addressing severe multicollinearity within the matrix of independent variables. In our model, changes in thermic effect of food and in hepatocellular lipid levels were chosen as the dependent variables and the metabolic variables (body weight, fat mass, visceral fat, and insulin resistance) represented the independent variables. The variability was separated into 2 independent components. The predictive component contained the variability in the metabolic variables, which was shared with changes in dependent variables, and the orthogonal component contained the variability shared within the metabolic variables. A detailed description of the orthogonal projections to latent structure model is available elsewhere.^28^ The statistical software SIMCA-P, version 11.5 (Umetrics AB) identified the number of relevant components using the prediction error sum of squares and also allowed the detection of multivariable nonhomogeneities and testing for multivariable normal distribution and homoscedasticity (constant variance). The statisticians (M.H, R.H.) were blinded to the interventions and group assignment. Results are presented as means with 95% CIs. Two-tailed tests were used to determine significance at the 5% level.

## Results

### Participant Characteristics

**Figure 1.**
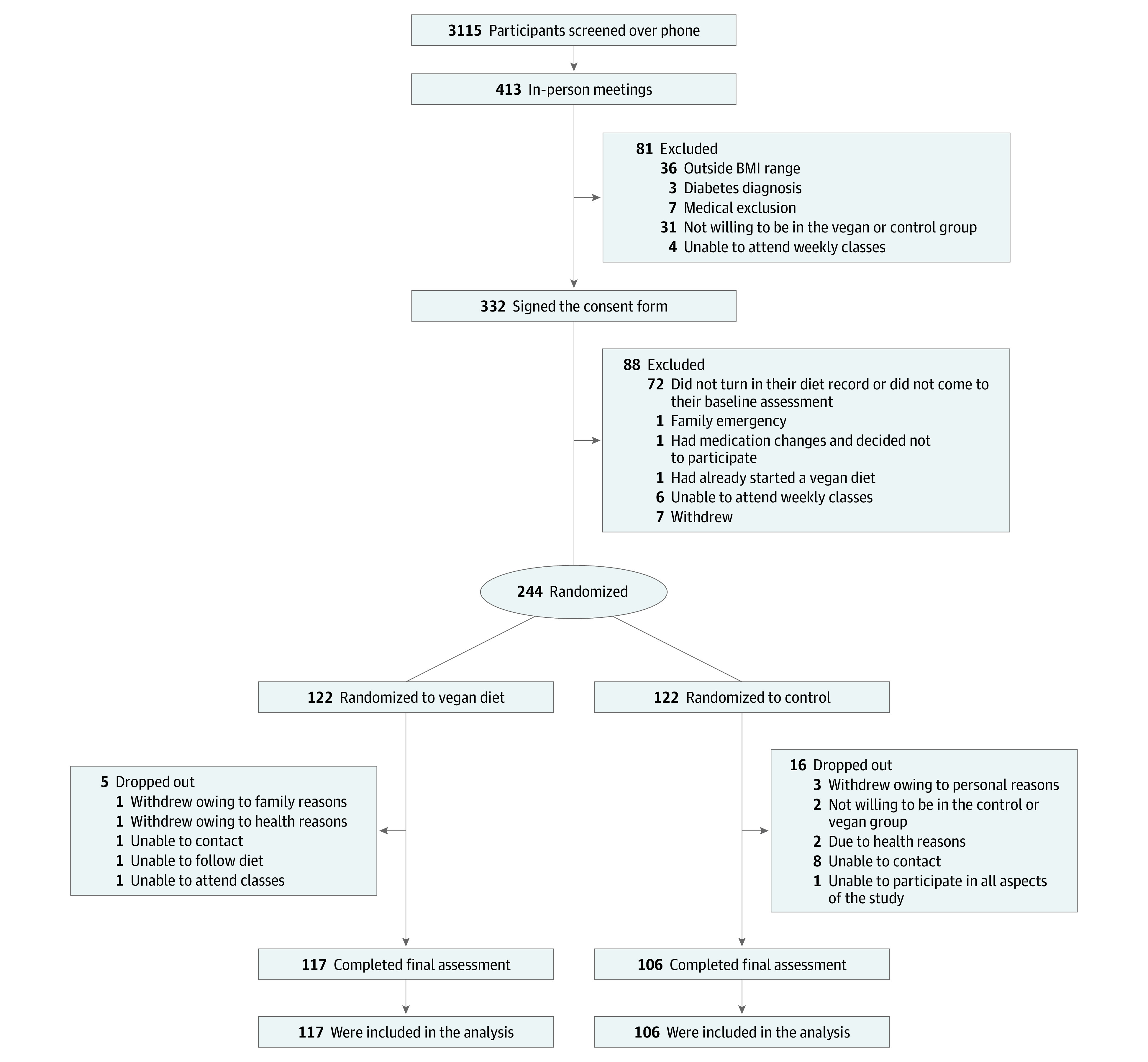
CONSORT Diagram of Participant Flow Through Trial

Of 3115 people screened by telephone, 244 met the participation criteria, signed the consent form and were randomly assigned to the intervention (n = 122) or control (n = 122) groups in a 1:1 ratio (Figure 1). The mean (SD) age of the intervention group was 53 (10) years compared with 57 (13) years in the control group (*P* = .01) (eTable 1 in Supplement 2). There were no other significant differences between the groups. Five intervention group and 16 control group participants dropped out, mostly for reasons unrelated to the study, leaving 222 (91.0%) individuals who completed the study. eTable 2 in Supplement 2 shows the baseline characteristics of those who completed the study and those who dropped out. There were no significant differences between these groups. The main outcomes are reported in Table 1. The treatment effects were largely unaffected by the adjustment for age and race/ethnicity (eTable 4 in Supplement 2). eTable 3 in Supplement 2 shows the characteristics of the subgroup that underwent magnetic resonance spectroscopy. This group had a lower BMI compared with the rest of the study population. The model adjusted for baseline BMI for magnetic resonance spectroscopy is presented in eFigure 2 in Supplement 2.

**Table 1.** Changes in Outcomes During the Study in the Low-Fat Vegan Dietary Intervention Group vs the Control Group

Outcome	Value, Mean (95% CI)							*P* value^a^
	Control group			Intervention group			Effect Size	
	Baseline	Week 16	Change	Baseline	Week 16	Change		
Dietary intake								
Energy intake, kcal/d	1793 (1670 to 1915)	1657 (1548 to 1766)	−135.8 (−250.7 to −20.8)^b^	1834 (1729 to 1940)	1344 (1260 to 1428)	−490.7 (−607.9 to −373.5)^c^	−354.9 (−519.0 to −190.8)	<.001
Fiber intake, g/d	23.9 (21.9 to 25.9)	23.3 (21.4 to 25.3)	−0.56 (−2.6 to 1.5)	24.1 (22.1 to 26.0)	34.6 (32.1 to 37.2)	10.6 (7.8 to 13.3)^c^	11.1 (7.8 to 14.5)	<.001
Cholesterol intake, mg/d	244.5 (211.4 to 277.6)	230.5 (196.1 to 264.9)	−14.0 (−51.7 to 23.7)	238.6 (212.3 to 265.0)	5.5 (3.8 to 7.3)	−233.1 (−259.4 to −206.8)^c^	−219.1 (−264.9 to −173.3)	<.001
Saturated fatty acids, g/d	22.9 (20.5 to 25.3)	20.5 (18.1 to 23.0)	−2.4 (−4.9 to 0.1)	23.6 (21.4 to 25.8)	5.1 (4.5 to 5.6)	−18.6 (−20.7 to −16.5)^c^	−16.2 (−19.4 to −13.0)	<.001
Monounsaturated fatty acids, g/d	27.9 (25.2 to 30.7)	25.4 (23.0 to 27.8)	−2.5 (−4.9 to −0.1)^b^	27.2 (25.3 to 29.1)	8.4 (7.6 to 9.2)	−18.8 (−20.7 to −16.9)^c^	−16.3 (−19.3 to −13.3)	<.001
Polyunsaturated fatty acids, g/d	19.1 (17.0 to 21.1)	18.0 (16.3 to 19.7)	−1.1 (−3.0 to 0.9)	18.4 (16.8 to 19.9)	9.5 (8.6 to 10.4)	−8.9 (−10.6 to −7.1)^c^	−7.8 (−10.4 to −5.2)	<.001
Physical activity, METs	2863 (2224 to 3502)	2153 (1605 to 2702)	−709.8 (−1346 to −73.9)^b^	2719 (1805 to 3633)	2114 (1619 to 2609)	−604.8 (−1388 to 178.6)	105 (−898 to 1108)	.84
Anthropometric variables and body composition								
Weight, kg	92.7 (90.0 to 95.3)	92.2 (89.4 to 94.9)	−0.5 (−1.0 to 0.1)	93.6 (91.0 to 96.1)	87.2 (84.9 to 89.6)	−6.4 (−7.0 to −5.7)^c^	−5.9 (−6.7 to −5.0)	<.001
BMI	33.6 (32.9 to 34.3)	33.9 (32.6 to 35.2)	0.3 (−0.7 to 1.3)	33.3 (32.6 to 34.0)	31.4 (30.5 to 32.4)	−1.9 (−2.5 to −1.3)^c^	−2.2 (−3.3 to −1.1)	<.001
Fat mass, kg	40.9 (39.1 to 42.8)	41.0 (39.0 to 42.9)	0.01 (−0.3 to 0.4)	40.6 (38.9 to 42.2)	36.5 (34.9 to 38.1)	−4.1 (−4.6 to −3.6)^c^	−4.1 (−4.7 to −3.5)	<.001
Lean mass, kg	49.5 (47.9 to 51.1)	48.9 (47.4 to 50.5)	−0.6 (−0.9 to −0.3)^c^	50.5 (49.0 to 51.9)	48.4 (47.1 to 49.8)	−2.1 (−2.4 to −1.8)^c^	−1.5 (−1.9 to −1.1)	<.001
VAT volume, cm^3^	1517 (1339 to 1695)	1510 (1324 to 1695)	−7.7 (−78.5 to 63.0)	1459 (1286 to 1632)	1243 (1096 to 1390)	−216.5 (−280.9 to −152.2)^c^	−208.8 (−303.7 to −113.7)	<.001
Hepatocellular lipids, %	3.3 (3.1 to 3.5)	3.6 (3.5 to 3.8)	0.3 (−0.5 to 1.2)	3.2 (3.0 to 3.4)	2.4 (2.3 to 2.5)	−0.8 (−1.5 to −0.1)^b^	−1.2 (−2.2 to −0.1)	.002
Intramyocellular lipids, %	1.5 (1.4 to 1.6)	1.7 (1.5 to 1.8)	0.13 (−0.05 to 0.21)	1.6 (1.5 to 1.7)	1.5 (1.4 to 1.6)	−0.1 (−0.2 to 0.05)	−0.3 (−0.4 to −0.1)	.03
Parameters of glucose control and insulin resistance								
HbA_1c_, DCCT, %	5.7 (5.6 to 5.8)	5.7 (5.6 to 5.8)	0.01 (−0.04 to 0.05)	5.6 (5.6 to 5.7)	5.6 (5.5 to 5.7)	−0.06 (−0.12 to −0.002)^b^	−0.07 (−0.1 to 0.01)	.07
Fasting plasma insulin level, pmol/L	78.9 (68.3 to 89.4)	103.5 (71.4 to 135.6)	23.6 (−5.0 to 54.3)	91.2 (79.9 to 102.5)	69.6 (56.9 to 82.3)	−21.6 (−35.9 to −7.3)^d^	−46.2 (−79.0 to −13.4)	.006
Fasting plasma glucose level, mmol/L	5.0 (4.7 to 5.4)	5.5. (5.4 to 5.7)	0.5 (0.2 to 0.8)^c^	5.2 (5.1 to 5.3)	5.1 (5.0 to 5.2)	−0.1 (−0.2 to 0.02)	−0.6 (−0.2 to −1.0)	.001
PREDIM	4.4 (4.1 to 4.7)	4.2 (3.9 to 4.5)	−0.2 (−0.4 to 0.04)	4.1 (3.8 to 4.3)	4.7 (4.4 to 5.0)	0.7 (0.5 to 0.9)^c^	0.9 (0.5 to 1.2)	<.001
HOMA	2.7 (2.3 to 3.2)	3.2 (2.4 to 4.0)	0.5 (−0.3 to 1.2)	3.2 (2.7 to 3.6)	2.3 (1.9 to 2.8)	−0.8 (−1.3 to −0.3)^c^	−1.3 (−2.2 to −0.3)	<.001
Lipid levels, mmol/L								
Total cholesterol	5.0 (4.7 to 5.2)	5.1 (4.9 to 5.3)	0.1 (−0.1 to 0.4)	5.2 (5.0 to 5.4)	4.7 (4.5 to 4.9)	−0.5 (−0.7 to −0.4)^c^	−0.6 (−0.9 to −0.4)	<.001
Triglycerides	1.3 (1.2 to 1.4)	1.3 (1.1 to 1.5)	−0.01 (−0.14 to 0.12)	1.2 (1.1 to 1.3)	1.4 (1.3 to 1.5)	0.2 (0.08 to 0.3)^c^	0.2 (0.03 to 0.4)	.02
HDL cholesterol	1.7 (1.5 to 1.9)	1.5 (1.4 to 1.6)	−0.2 (−0.4 to −0.02)^b^	1.6 (1.5 to 1.6)	1.4 (1.3 to 1.4)	−0.2 (−0.3 to −0.1)^c^	0.01 (−0.2 to 0.2)	.93
LDL cholesterol	2.9 (2.6 to 3.1)	3.0 (2.9 to 3.2)	0.07 (−0.02 to 0.2)	3.1 (3.0 to 3.3)	2.7 (2.5 to 2.9)	−0.4 (−1.0 to −0.3)^c^	−0.5 (−0.8 to −0.3)	<.001

Abbreviations: BMI, body mass index (calculated as weight in kilograms divided by height in meters squared); DCCT, Diabetes Control and Complications Trial; HbA_1c_, glycated hemoglobin; HDL, high-density lipoprotein; HOMA, homeostasis model assessment; LDL, low-density lipoprotein; METs, metabolic equivalents; PREDIM, predicted insulin sensitivity index; VAT, visceral adipose tissue.

SI conversion factors: To convert plasma insulin level to μIU/mL, divide by 6.945; plasma glucose level to mg/dL, divide by 0.0555; and lipid levels to mg/dL, divide by 0.0259.

^a^ *P* values are for the interaction between group and time assessed by repeated measures analysis of variance.

^b^ *P* < .05 for within-group changes from baseline assessed by paired comparison *t* tests.

^c^ *P* < .001 for within-group changes from baseline assessed by paired comparison *t* tests.

### Dietary Intake and Physical Activity

Self-reported energy intake decreased in both groups but more so in the intervention group (treatment effect, −354.9 kcal/d; 95% CI, −519.0 to −190.8 kcal/d; *P* < .001) (Table 2). In the intervention group, mean intakes of carbohydrate and fiber increased, whereas mean fat, protein, and cholesterol intake decreased. These values did not change significantly in the control group. Physical activity decreased slightly in both groups (−709.8 metabolic equivalents [95% CI, −1346 to −73.9 metabolic equivalents] in the control group and −604.8 metabolic equivalents [95% CI, −1388 to −178.6 metabolic equivalents] in the intervention group; between-group *P* = .84).

**Table 2.** Relationship Between Changes in Thermic Effect of Food and the First Predictive Component as Evaluated by the OPLS Model

Variable	OPLS predictive component				Multiple regression	
	Component loading^a^	*t* Statistic	*R*^b^	*P* value for *R*	Regression coefficient	*t* Statistic
Matrix X						
Baseline BMI	0.191	2.46	0.209	<.05	−0.015	−0.33
Baseline fat mass	0.256	2.89	0.283	<.05	−0.014	−0.28
Baseline TEF	−0.850	−11.96	−0.938	.005	−0.505	−5.69^c^
Change in PREDIM	0.324	2.41	0.359	<.05	0.105	1.37
Change in fat mass	−0.271	−2.59	−0.301	<.05	−0.122	−1.55
Matrix Y						
Change in TEF	1.000	5.27	0.540	.003	NA	NA

Abbreviations: BMI, body mass index; NA, not applicable; OPLS, orthogonal projections to latent structure; PREDIM, predicted insulin sensitivity index; TEF, thermic effect of food.

^a^ The explained variability was 29.2% (24.3% after cross-validation).

^b^ Component loadings expressed as a correlation coefficients with predictive component.

### Body Weight, Body Composition, and Blood Lipid Levels

Mean body weight decreased by 6.4 kg in the intervention group compared with 0.5 kg in the control group (treatment effect, −5.9 kg; 95% CI, −6.7 to −5.0; interaction between group and time, *P* < .001). This difference was largely attributable to a reduction in body fat, as noted by significant decreases in fat mass and visceral fat volume in the intervention group participants. Total and low-density lipoprotein cholesterol levels decreased by 0.5 mmol/L and 0.4 mmol/L (to convert to milligrams per deciliter, divide by 0.0259), respectively, in the intervention group, with no significant changes in the control group (0.1 mmol/L and 0.07 mmol/L, respectively) (*P* < .001 for both).

### Insulin Sensitivity

Fasting plasma insulin concentration decreased by 21.6 pmol/L (to convert to micro-IU per milliliter, divide by 6.945) in the intervention group, with no significant change in the control group (23.6 pmol/L; 95% CI, −5.0 to 54.3; between-group *P* = .006). The homeostasis model assessment index (a measure of insulin resistance) decreased significantly (−1.3; 95% CI, −2.2 to −0.3; *P* < .001), and PREDIM (a measure of insulin sensitivity) increased significantly in the intervention group (0.9; 95% CI, 0.5-1.2; *P* < .001); neither changed significantly in the control group (Table 2). Within the intervention group, the change in PREDIM correlated negatively with the change in body weight (*r* = −0.43; *P* < .001).

### Postprandial Metabolism

**Figure 2.**
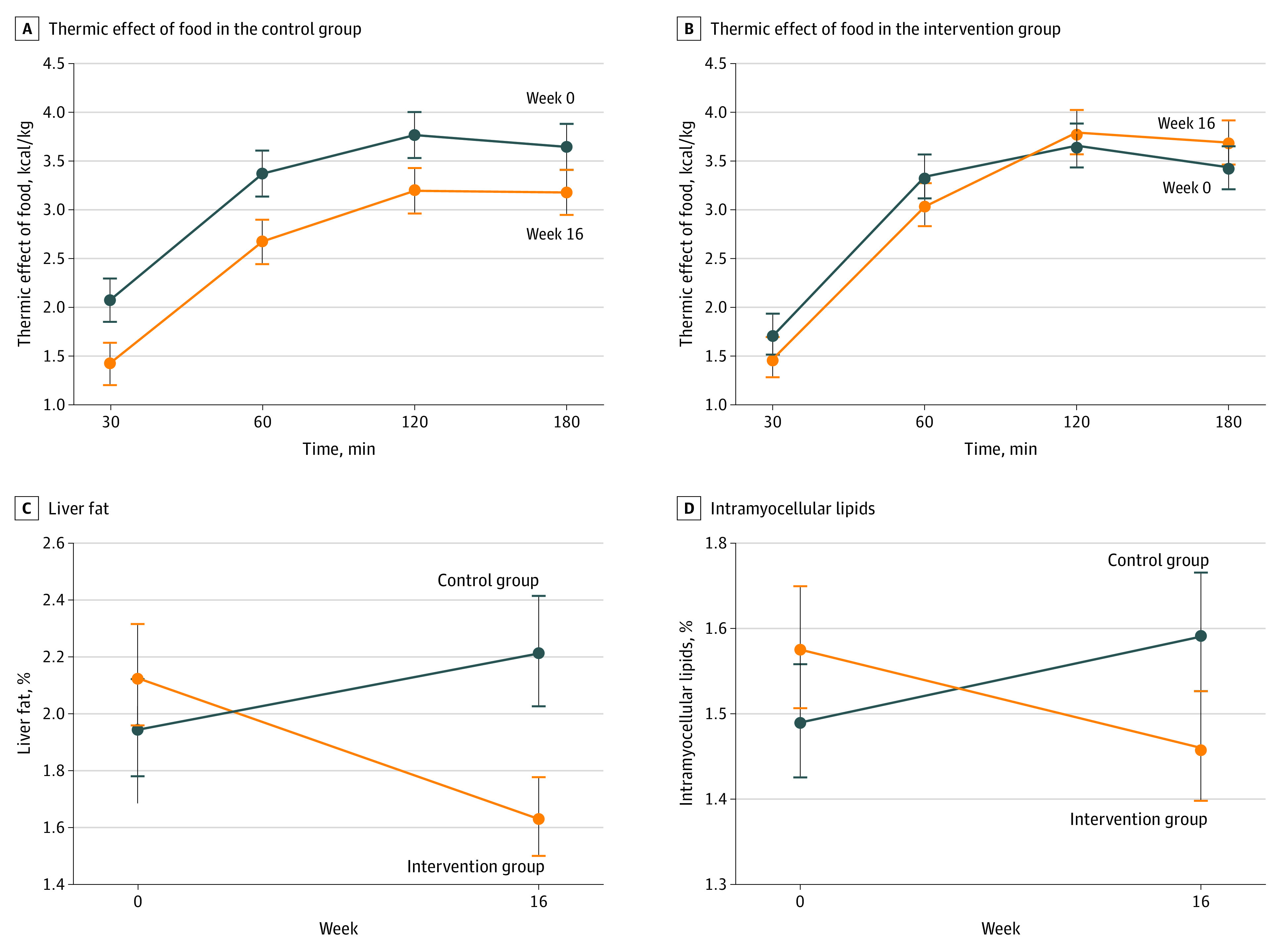
Changes in the Thermic Effect of Food, Liver Fat, and Intramyocellular Lipid Levels in the Intervention and Control Groups Whiskers represent 95% CIs.

Postprandial energy expenditure (the thermic effect of food) increased by 18.7% (95% CI, 4.4%-22.3%) in the intervention group from baseline to 16 weeks and did not change significantly in the control group (14.1%; 95% CI, 6.5%-20.4%) (interaction between group and time, *P* < .001) (Figure 2A). The *F* values were as follows: group, *F* = 1.7 (*P* = .19); week, *F* = 15.4 (*P* < .001); time, *F* = 122.4 (*P* < .001); group × week, *F* = 11.9 (*P* < .001); group × time, *F* = 1.1 (*P* = .35); week × time, *F* = 1.38 (*P* = .25). The results were similar in models adjusted for age and race/ethnicity (eFigure 1 in Supplement 2). Within the intervention group, the change in thermic effect of food did not correlate significantly with changes in body weight (*r* = −0.15; *P* = .09), PREDIM (*r* = 0.06; *P* = .54), energy intake (*r* = 0.01; *P* = .90), or fiber consumption (*r* = 0.07; *P* = .48). In both groups combined, changes in thermic effect of food correlated negatively with changes in fat mass (*r* = −0.30; *P* < .05) and positively with changes in PREDIM (*r* = 0.36; *P* < .05). That is, as fat mass decreased and insulin sensitivity improved, postprandial metabolism increased (Table 2).

A linear regression model for changes in reported energy intake and body weight showed that every 100 kcal/d change in energy intake was associated with a 0.15 kg change in body weight (eFigure 3 in Supplement 2). The mean (SD) reported energy reduction of 355 (617) kcal in the intervention group compared with the control group would therefore be associated with a mean (SD) weight loss of 0.53 (4.4) kg. For changes in postprandial energy expenditure and body weight, every change in postprandial energy expenditure of 10 000 U in area under the curve was associated with a change in body weight of 0.48 kg (eFigure 3 in Supplement 2). The mean (SD) decrease in postprandial energy expenditure of 8588 (34 020) U of area under the curve was associated with an mean (SD) weight loss of 0.41 (2.8) kg.

### Hepatocellular and Intramyocellular Lipid Levels

In the 44 participants for whom hepatocellular and intramyocellular lipid levels were quantified, baseline hepatocellular lipid content was generally in the normal range.^29,30^ Nonetheless, hepatocellular lipid content decreased in the intervention group by 34.4% (from a mean [SD] of 3.2% [2.9%] to 2.4% [2.2%]; *P* = .03) and remained unchanged in the control group (from a mean [SD] of 3.3% [4.3%] to 3.6% [4.7%]) (group, *F* = 3.1 [*P* = .09]; week, *F* = 1.27 [*P* = .27]; group × week, *F* = 10.8 [*P* = .002]) (Figure 2B). Results were similar in models adjusted for age and race/ethnicity (eFigure 1 in Supplement 2) and for baseline BMI (eFigure 2 in Supplement 2).

Within the intervention group, the decrease in hepatocellular lipid levels was significantly associated with change in body weight (*r* = 0.42; *P* = .04) but not with changes in reported energy intake (*r* = 0.24; *P* = .27) or fiber consumption (*r* = 0.07; *P* = .76). In both groups combined, changes in hepatocellular lipid levels correlated negatively with changes in PREDIM (*r* = −0.47; *P* < .05). That is, as hepatocellular lipid level decreased, insulin sensitivity increased. Changes in hepatocellular lipid levels correlated positively with changes in body weight (*r* = 0.91; *P* < .01), BMI (*r* = 0.90; *P* < .01), fat mass (*r* = 0.91; *P* < .01), and visceral fat (*r* = 0.80; *P* < .01) (Table 3).

**Table 3.** Relationship Between Changes in Liver Fat and the First Predictive Component as Evaluated by OPLS Model

Variable	OPLS predictive component				Multiple regression		
	Component loading^a^	*t* Statistic	*R*^**b**^	*P* value for *R*	Regression coefficient	*t* Statistic	*P* value for *t*
Matrix X							
Control group	0.339	9.88	0.795	.004	0.069	3.69	.007
Intervention group	−0.339	−9.88	−0.795	.004	−0.069	−3.69	.007
Baseline PREDIM	0.214	8.89	0.498	.003	0.038	4.99	.005
Baseline HOMA	−0.218	−2.71	−0.509	<.05	−0.060	−2.07	<.05
Baseline weight	−0.228	−2.18	−0.535	<.05	−0.065	−2.19	<.05
Baseline fat mass	−0.221	−2.18	−0.518	<.05	−0.058	−2.42	<.05
Change in PREDIM	−0.199	−2.35	−0.468	<.05	−0.021	−2.54	<.05
Change in weight	0.388	14.13	0.910	.005	0.079	5.47	.005
Change in BMI	0.384	13.92	0.901	.005	0.077	5.64	.003
Change in fat mass	0.389	21.06	0.911	.002	0.078	5.87	.006
Change in visceral fat	0.341	8.23	0.798	.007	0.060	2.63	<.05
Matrix Y							
Change in liver fat	1.000	4.66	0.495	.009	NA	NA	NA

Abbreviations: BMI, body mass index; HOMA, homeostasis model assessment; OPLS, orthogonal projections to latent structure; NA, not applicable; PREDIM, predicted insulin sensitivity index.

^a^ Explained variability was 24.5% (20.8% after cross-validation).

^b^ Component loadings expressed as a correlation coefficients with predictive component.

Changes in intramyocellular lipid levels were not statistically significant in within-group comparisons, but owing to the opposite trends, the treatment effect was significantly decreased in the intervention group by 10.4%, from a mean (SD) of 1.6 (1.1) to 1.5 (1.0) (*P* = .03) (group, *F* = 4.7 [*P* = .04]; week, *F* = 0.02 [*P* = .88]; group × week, *F* = 5.1 [*P* = .03]) (Figure 1C). Within the intervention group (n = 23), changes in both hepatocellular and intramyocellular lipid levels correlated with changes in insulin resistance, as measured by the homeostasis model assessment index (both *r* = 0.51; *P* = .01). In both groups combined, changes in intramyocellular lipid levels correlated positively with changes in fat mass (*r* = 0.51; *P* < .05) and homeostasis model assessment index score (*r* = 0.52; *P* < .05). That is, as fat mass decreased, intramyocellular lipid levels and insulin resistance decreased.

## Discussion

In this trial, the dietary intervention reduced body weight, apparently owing to its tendency to reduce energy intake and increase postprandial energy expenditure. The intervention also improved glycemic control and reduced insulin concentrations, owing in part to reduced lipid accumulation in liver and muscle cells and thus reduced insulin resistance in these organs.

The intervention diet’s effect on weight and insulin action are clinically important. Hepatocellular and intramyocellular lipids play central roles in hepatic and muscle insulin resistance, respectively, and in type 2 diabetes. A 16-week diet of 1200 kcal per day resulted in a moderate weight loss of approximately 8 kg, which was sufficient to normalize liver lipid content and fasting plasma glucose concentrations as well as reverse hepatic insulin resistance in patients with obesity and type 2 diabetess.^31^ A potential mechanism explaining the improvement in insulin sensitivity is the reduction in intracellular diacylglycerol levels, which reduce insulin signaling in liver and muscle, leading to tissue-specific insulin resistance.^22,32,33^

The effects of the dietary intervention on hepatocellular and intramyocellular lipid levels and insulin sensitivity—the presumed basis for the improved glycemic control—has not previously been quantified in clinical trials. Energy restriction has been shown to reduce intramyocellular and hepatocellular lipid levels and improve glycemic control in healthy young individuals without diabetes.^26,34,35^ In young, lean individuals with insulin resistance, a hypocaloric diet (approximately 1200 kcal) led to a mean weight loss of 4.1 kg and a 30% reduction of intramyocellular lipids during a 9-week intervention.^26^ In contrast, the intervention diet in the present study did not restrict energy intake but nonetheless led to 34% and 10% reductions in hepatocellular and intramyocellular lipid levels, respectively. The reductions in hepatocellular and intramyocellular lipid levels correlated with the reduction in fat mass, consistent with prior studies.^26,36,37^

The present finding that the increase in thermic effect of food was associated with decreased fat mass and increased insulin sensitivity confirm the findings of previous research.^38,39^ The increased insulin sensitivity may have contributed to the increased postprandial metabolism. In addition, increased postprandial metabolism may have promoted further reduction in fat mass and an increase in insulin sensitivity.

Despite the ad libitum diet, the participants in the intervention group reduced their energy intake, consistent with many previous trials using vegan diets. This not only contributes to weight loss but also may have contributed to the decrease in hepatocellular triglyceride content.^31^

Postprandial metabolism is influenced by meal composition.^40,41,42,43^ In the present study, however, the test meal was identical for all study phases. These results suggest that the increased postprandial thermogenesis was attributable to improved insulin sensitivity.

### Strengths and Limitations

This study has several strengths. The randomized parallel design in which all participants within each cohort began the study simultaneously controlled for seasonal diet fluctuations. The study duration provided sufficient time for adaptation to the diet. Physiologic stimulation by a standard mixed meal permitted quantification of insulin sensitivity and insulin secretion during a physiologic perturbation. Measurement of visceral, hepatocellular, and intramyocellular lipid levels, in addition to the detailed assessment of the thermic effect of food, are also strengths. The low attrition suggests that the intervention was acceptable.

The study also has limitations. Self-reports of dietary intake have well-known limitations.^44^ However, it is reassuring that the reported diet changes were paralleled by changes in weight and plasma lipid levels. Health-conscious participants may not be representative of the general population but may be representative of a clinical population seeking help for weight problems or type 2 diabetes. We followed the participants for 16 weeks and were not able to estimate the effects of the diet over a longer period. In addition, the study design did not allow separation of the specific effects of the low-fat vegan diet from the weight loss it causes.

## Conclusions

This randomized clinical trial found that a low-fat plant-based dietary intervention reduces body weight by reducing energy intake and increasing postprandial metabolism, apparently owing to increased insulin sensitivity resulting from reduced hepatocellular and intramyocellular fat. This intervention may be an effective treatment for overweight adults.

## Notes

Supplement 1. **Trial Protocol**

Supplement 2. **eTable 1.** Baseline characteristics of the study population **eTable 2.** Baseline characteristics of the study population, comparing study completers and drop-outs **eTable 3.** Baseline characteristics of the study population, comparing the subsample undergoing magnetic resonance spectroscopy (MRS) with the rest of the study population **eTable 4.** Treatment effects for the main outcomes, adjusted for age and race **eFigure 1.** Changes in the thermic effect of food, liver fat, and intramyocellular lipids after adjustment for race and age **eFigure 2.** Changes in liver fat and intramyocellular lipids after adjustment for baseline BMI **eFigure 3.** Linear regression model for changes in energy and body weight and postprandial energy expenditure and body weight

Supplement 3. **Data Sharing Statement**
