# Clarification of Role of Funder Statement

**DOI:** 10.1001/jamanetworkopen.2020.35088

**Published:** 2021-01-07

In the Original Investigation titled “Effect of a Low-Fat Vegan Diet on Body Weight, Insulin Sensitivity, Postprandial Metabolism, and Intramyocellular and Hepatocellular Lipid Levels in Overweight Adults: A Randomized Clinical Trial,”^1^ published on November 30, 2020, the Role of the Funder/Sponsor statement was updated to the following: “The Yale Diabetes Center had no role in the design and conduct of the study; collection, management, analysis, and interpretation of the data; preparation, review, or approval of the manuscript; and decision to submit the manuscript for publication. Drs Kahleova, Alwarith, and Rembert, as employees of Physicians Committee for Responsible Medicine, were involved in the design and conduct of the study; collection, management, analysis, and interpretation of the data; preparation, review, or approval of the manuscript; and decision to submit the manuscript for publication. The research team of the Physicians Committee for Responsible Medicine had full autonomy in all aspects of the study.” This article has been corrected.
