# Errors in Abstract and Main Results

**DOI:** 10.1001/jamanetworkopen.2021.0550

**Published:** 2021-02-01

In the Original Investigation titled “Effect of a Low-Fat Vegan Diet on Body Weight, Insulin Sensitivity, Postprandial Metabolism, and Intramyocellular and Hepatocellular Lipid Levels in Overweight Adults: A Randomized Clinical Trial,”^1^ published November 30, 2020, corrections were made to clarify that the effect of food increased in the intervention group from baseline to 16 weeks and did not change significantly in the control group as indicated by the between-group difference effect size. This article has been corrected.
