# Error in the Results and eTable 2 in Supplement 2

**DOI:** 10.1001/jamanetworkopen.2021.15510

**Published:** 2021-05-27

In the Original Investigation titled “Effect of a Low-Fat Vegan Diet on Body Weight, Insulin Sensitivity, Postprandial Metabolism, and Intramyocellular and Hepatocellular Lipid Levels in Overweight Adults: A Randomized Clinical Trial,”^1^ published November 30, 2020, in the first paragraph of the Results and in eTable 2 in Supplement 2, the number of participants who completed the study was changed from 222 to 223. This article has been corrected.^1^
