# Supplementary material for: Effect of a Low-Fat Vegan Diet on Body Weight, Insulin Sensitivity, Postprandial Metabolism, and Intramyocellular and Hepatocellular Lipid Levels in Overweight Adults: A Randomized Clinical Trial
Source: JAMA Netw Open. 2020 Nov 30;3(11):e2025454. doi: 10.1001/jamanetworkopen.2020.25454 (PMC7705596; doi:10.1001/jamanetworkopen.2020.25454)
Supplement: Supplement 1. — Trial Protocol [file jamanetwopen-e2025454-s001.pdf]

A Randomized, Controlled Trial on Diet, Insulin Sensitivity, and Postprandial Metabolism

Summary

This randomized, controlled trial aims to elucidate the mechanisms by which a plant-based dietary intervention causes weight loss. Using a low-fat, plant-based diet for 16 weeks, along with an untreated control for comparison, the study will measure changes in insulin sensitivity, postprandial metabolism, and intracellular lipid, and assess their associations with changes in body weight.

1. SPECIFIC AIMS AND OVERVIEW

1.1. Specific Aims

Specific Aim 1. This study tests the hypothesis that weight changes associated with a low-fat plant-based diet are, in part, the result of increased postprandial metabolism (thermic effect of food).

Specific Aim 2. This study tests the hypothesis that increased postprandial metabolism in response to a diet intervention is the result of increased insulin sensitivity.

Specific Aim 3. This study conducts a pilot substudy to test the hypothesis that changes in insulin sensitivity observed in response to a diet intervention correlate with changes in intramyocellular and/or intrahepatocellular lipid.

1.2. Protocol Overview

In a 16-week trial, overweight adults will be randomly assigned to two groups. Changes in insulin sensitivity, postprandial metabolism, and body weight will be the primary dependent variables.

The Diet Group will be asked to follow a low-fat, vegan diet and will receive weekly classes and support.

The Control Group will be asked to make no changes in diet or exercise for 16 weeks, but will be instructed in the intervention diet at the study’s conclusion.

1.3. Investigative Team

The project will be conducted by investigators from the Physicians Committee for

Responsible Medicine (PCRM), a nonprofit 501(c)(3) organization located at 5100 Wisconsin Avenue, NW, Washington DC 20016, which conducts nutrition-related research. Its medical, nutrition, and research staff will oversee participant recruitment, screening, group assignment, nutrition teaching and monitoring, and data collection and analysis. Recruitment interviews, the dietary intervention, and most assessments will take place at its offices.

Researchers from the Department of Internal Medicine, Yale University School of Medicine, will conduct MR spectroscopy studies for intracellular lipid.

2. BACKGROUND AND SIGNIFICANCE

Excess body weight is a major contributor to many health problems, including diabetes, cardiovascular disease, orthopedic problems, and certain forms of cancer. In epidemiologic studies, individuals following vegan diets tend to have significantly lower body weights, compared with individuals following other dietary patterns.[1] In studies of overweight individuals, the adoption of a low-fat plant-based diet predictably reduces body weight, even in the absence of any specified limitation on energy intake.[2]

The mechanisms by which plant-based diets reduce body weight are not entirely clear. Previous studies have identified two possible explanations:[3] First, to the extent that vegan diets are low in fat and high in fiber, they have a relatively low energy density, which reduces energy intake. Second, a low-fat vegan diet may increase postprandial metabolism (the thermic effect of food). These observations suggest that the diet leads to weight loss by (1) reducing energy intake and (2) increasing postprandial energy output.

A prior study[3] including 64 overweight postmenopausal women randomly assigned to a low-fat vegan diet or a comparison diet based on the guidelines of the National Cholesterol Education Program for 14 weeks found that the vegan diet led to significantly greater weight loss (5.8 kg for the vegan group, compared with 3.8 kg for the comparison group). The vegan diet group also had a 16% increase in postprandial metabolism and an increase in insulin sensitivity that was significant within group, although not between groups. However, because the comparison group used an active diet and there was no untreated control group, that study was not able to show the degree to which a plant-based diet influences energy expenditure, compared with untreated participants.

Insulin resistance has been shown to be related to fat accumulation within muscle cells (intramyocellular lipid) and liver cells (intrahepatocellular lipid).[4] The above findings suggest the possibility that low-fat, vegan diets reduce the quantity of lipid stored within these cells, which, in turn, improves insulin sensitivity.

Some evidence suggests that the accumulation of intracellular fat may be responsive to diet. In a 2012 study at Yale University, 7 lean, young individuals who were known to be insulin-resistant and who had parents with type 2 diabetes underwent a hypocaloric (1200 kcal/d) diet for 9 weeks, leading to an average weight loss of 4.1 ± 0.6 kg. During

this intervention period, average intramyocellular lipid fell approximately 30%, from 1.1 ± 0.2% to 0.8 ± 0.1%.[5]

High-fat diets appear to downregulate the genes required for mitochondrial oxidative phosphorylation in skeletal muscle and increase intramyocellular lipid.[6] In contrast, a case-control study found that soleus muscle intramyocellular lipid concentrations were significantly lower in a group of 21 vegans, compared with 25 omnivores.[7]

In research studies, the acceptability of plant-based diets appears to be similar to that of other therapeutic diets over both the short and long term, as indicated by rates of retention, diet adherence, and diet acceptance questionnaires.[8-11] If a plant-based diet increases postprandial metabolism, its use for the prevention and management of weight disorders and related health problems will have a more solid rationale. This is especially important given that weight problems are widespread and there is a great deal of confusion among the public regarding which diet method to select.

3. RESEARCH DESIGN, RECRUITMENT, AND ASSESSMENTS

3.1. Overview of Research Design

In a randomized, controlled trial, we will test the effects of a low-fat, plant-based diet on insulin sensitivity, postprandial metabolism, and body weight in overweight adults over a 16-week period, using for comparison an untreated control. A substudy will examine effects on intracellular lipid.

3.2. Key Personnel

Key personnel include:

Neal D. Barnard, MD, FACC, Principal Investigator, is an Adjunct Associate Professor of Medicine at the George Washington University and President of PCRM. He has been the Principal Investigator of several clinical trials, as noted herein.

Hana Kahleova, MD, PhD, is an endocrinologist and Director of Clinical Research at PCRM. She has been involved in several clinical trials in diabetes and insulin resistance.

Susan Levin, MS, RD; Karen Smith, RD, and Maggie Neola, RD, are Registered Dietitians at PCRM who provide nutrition instruction and participate in clinical assessments.

Francesca Valente and Rosendo Flores coordinate clinical research studies at PCRM.

Richard Holubkov, PhD, is a biostatistician with the University of Utah, who works with PCRM on contract.

Gerald I. Shulman, MD, PhD, FACP, MACE, is a Professor of Medicine, Cellular and Molecular Physiology and The Howard Hughes Medical Institutes at Yale University School of Medicine.

Kitt Falk Petersen, MD, is a Professor of Medicine at Yale University School of Medicine.

3.3. Recruitment and Screening Procedures

Volunteers will be recruited through notices placed in waiting rooms of physicians' offices, letters sent to patients of medical practitioners, and advertisements placed in newspapers, on radio, and in buses in the Washington, DC, area, as well as social media postings. (**Appendix 1**)

Volunteers' initial calls will be directed to PCRM, where research staff will screen participants using a telephone screening script **(Appendix 2).** Research staff will explain the study, review participation criteria, and inquire about other motivations for volunteering, filling out a paper interview screening form for each person who calls. Volunteers who satisfy the participation criteria will be scheduled for group and/or individual information sessions. The names/identifiable information of volunteers who do not satisfy participation criteria will be destroyed (shredded) immediately. For these individuals, the research team will retain only de-identified demographic information and the reason for exclusion, for purposes of evaluating participation statistics.

At the group and/or individual information sessions (some volunteers may attend individually; others may be seen in groups), the investigators and research staff will explain the study and its scientific basis in detail and review participation criteria in simple, nontechnical terms. They will also provide instruction on filling out a diet record. Additional content will be determined by questions raised by volunteers, and may relate to study logistics, the recruitment process, the content of the vegan diet and ease of following it, clinical assessments, or the weekly classes. To protect patient privacy, volunteer names will not be used at these meetings. Volunteers will have a chance to ask questions about the study and the informed consent process in private, and each volunteer will meet in private with study personnel at the conclusion of the group session, even if he or she has no questions. Volunteers who choose to complete the informed consent document **(Appendix 3)** will also be asked to complete a contact information form **(Appendix 4)** and a general medical history form **(Appendix 5)**. Volunteers will be assigned identification numbers in the order in which they complete the informed consent document. These numbers will be used in place of identifying information for purposes of data collection, assessment, and analysis. During the screening process, prospective volunteers will be provided the study coordinator's phone number and contact information in order to be able to reschedule and cancel their appointments, if necessary.

Prospective volunteers will then be asked to complete a practice 2-day dietary record to demonstrate their ability to track nutrient intake for research purposes. A letter will be

sent to the participants to remind them to complete their 2-day diet record with instructions (**Appendix 6**). When completed, these records will be reviewed for completeness by a registered dietitian.

Volunteers who have completed the informed consent process and practice dietary records and meet the study participation criteria will be asked to schedule individual appointments for baseline assessments. There, they will be asked to fill out new 3-day dietary records. Volunteers can either submit questionnaires online, print, scan and email them, or send them through regular mail. Those who satisfactorily complete the baseline assessments and 3-day dietary records will be enrolled in the study.

The cost of all tests and procedures will be covered by PCRM. If any examination or test reveals that a participant has a medical condition that requires additional diagnostic tests or treatment, research staff will advise the participant of that fact, but will not provide such additional diagnostic tests or treatment. There is no cost for the weekly group sessions. Parking will be free during participants' visits to the PCRM office for assessments, meetings, and group sessions.

3.4. Inclusion and Exclusion Criteria

Inclusion criteria are as follows:

1. Men and women age ≥18 years of age
2. Body mass index 28-40 kg/m^2

Exclusion criteria are as follows:

1. Diabetes mellitus, type 1 or 2, history of diabetes mellitus or of any endocrine condition that would affect body weight, such as thyroid disease, pituitary abnormality, or Cushing's syndrome
2. Smoking during the past six months
3. Alcohol consumption of more than 2 drinks per day or the equivalent, episodic increased drinking (e.g., more than 2 drinks per day on weekends), or a history of alcohol abuse or dependency followed by any current use
4. Use of recreational drugs in the past 6 months
5. Use within the preceding six months of medications that affect appetite or body weight, such as estrogens or other hormones, thyroid medications (unstable dose within the preceding 6 months), systemic steroids, antidepressants (tricyclics, MAOIs, SSRIs), antipsychotics, lithium, anticonvulsants, appetite suppressants or other weight-loss drugs, herbs for weight loss or mood, St. John's wort, ephedra, beta blockers
6. Pregnancy or intention to become pregnant during the study period
7. Unstable medical or psychiatric illness
8. Evidence of an eating disorder
9. Likely to be disruptive in group sessions

10. Already following a low-fat, vegan diet
11. Lack of English fluency
12. Inability to maintain current medication regimen
13. Inability or unwillingness to participate in all components of the study
14. Intention to follow another weight-loss method during the trial

3.5. Group Assignment

Participants will be told that, if accepted, they will be assigned either to a Diet Group or a Control Group. Accepted volunteers will be assigned to these groups using a computer-generated random-number table. Because assignment will be done simultaneously within each replication, allocation concealment is unnecessary.

3.6. Clinical Assessments

The following determinations will be made at baseline and 16 weeks, except as noted:

Assessments of Dietary Intake and Physical Activity

3-day dietary record. A 3-day dietary record will be used to assess macro- and micronutrient intakes. Records will be analyzed using Nutrition Data System for Research software version 2016, developed by the Nutrition Coordinating Center (NCC), University of Minnesota, Minneapolis, MN, US, by a registered dietitian certified by the NCC. Random 24-hour dietary recalls will be conducted by registered dietitians to determine compliance but will not be part of the final nutrient analysis.

The **International Physical Activity Questionnaire** short form assesses recent physical activity patterns. The method is highly reliable; an assessment of test-retest repeatability produced a correlation of 0.8.[12] **(Appendix 7)**

Assessments of Physical Health, Weight, and Metabolism

General status, symptoms, and medication accounting. Participants will be asked to report changes in their health and medication use.

Height. Height will be measured at baseline (only) with participants standing barefoot with their backs to a wall-mounted stadiometer and heels against the wall, recorded to the nearest 0.5 cm.

Body weight. With participants wearing light, indoor clothing but without shoes, body weight will be measured to the nearest 0.1 kg, using a digital scale. Body weight will also be assessed at each weekly group session for the Diet Group, but only data from weeks 0, 8, and 16 will be included in the analysis.

Comprehensive Metabolic Panel. These values will be evaluated at baseline only.

Serum cholesterol and triacylglycerol concentrations and h**emoglobin A1c** will be measured using standard methods.

The following measures will be assessed at weeks 0 and 16:

Glucose Tolerance and Insulin Sensitivity. An oral glucose tolerance test will be performed for three hours after an overnight fast. (Matsuda 1999)

Resting Energy Expenditure (REE). Participants will be asked to report to the laboratory within 60 minutes of waking and after a 12-hour fast. Following 30 minutes of quiet rest in a dimly lit room, pulse, respiratory rate, and body temperature will be measured. REE will be measured for 20 minutes through indirect calorimetry (Cosmed Quark RMR, Chicago, IL) utilizing a ventilated hood system. The laboratory temperature will be maintained at 23 degrees C throughout, and precautions will be taken to minimize any disturbances that could affect the metabolic rate.

For premenopausal women, measures will be timed so as to occur in the luteal phase of the menstrual cycle.

Postprandial metabolism (thermic effect of food, TEF). After the REE determination, participants will be given a 720-kilocalorie test meal (Sustacal, Mead Johnson, Evansville, IN) to be ingested within 10 minutes. Metabolic rate will be measured in the same manner as above for 30 minutes at 2 and 4 hours postingestion.

Body Composition. Body composition will be measured by dual energy x-ray absorptometry (Lunar iDXA, GE Healthcare; Madison, WI) with Encore® 2005 v.9.15.010 software. The iDXA can measure body composition with low X ray exposure and short scanning time. The iDXA unit will be calibrated daily using the GE Lunar calibration phantom, and a trained operator will perform all scans following standard protocol for participant positioning. The iDXA is equipped with the CoreScan module (GE Healthcare, Madison, WI), which can also provide an estimate of visceral adipose tissue volume and mass.

Intramyocellular and Hepatic Lipid Content. A subset of participants will be selected for MR spectroscopy studies quantifying hepatic lipid and/or intramyocellular and/or contents in order to provide data regarding possible causal relationships between dietary changes, ectopic lipid, and insulin sensitivity. Selected individuals with varying degrees of insulin-resistance in both groups will be assessed before and after the intervention period. These MRS studies will take place at the Magnetic Research Center at Yale University School of Medicine, New Haven, CT.

Intramyocellular and hepatic lipid contents will be measured using ^{1}H MRS at 4T (Bruker).[13] After safety procedures including completion of the Yale Magnetic Research Center Safety Questionnaire, changing into scrubs and passing through the metal detector, the participants will positioned on their back on the bed, which slides into the

MRS instrument. For the leg lipid measurements the right leg will be positioned in a holder with the calf muscle over a receiver coil.

Muscle lipid content will be measured in the soleus muscle using an 8.5-cm diameter circular ^{13}C surface coil with twin, orthogonal circular 13-cm ^{1}H quadrature coils. The probe will be as tuned and matched and scout images of the lower leg will be obtained to ensure correct positioning of the participant and to define an adequate volume for localized shimming using the FASTMAP procedure.[14] The measurement will take approximately 30 minutes.

After the lipid measurements in the leg a receiver coil embedded in a plastic plate will be positioned on the side of the abdomen over the liver and strapped in place with velco straps. The position of the coil will be confirmed with MR images and the location of the lipid measurement within the coil will be determined from these MR images.Liver triglyceride content will be measured by ^{1}H respiratory-gated STEAM spectroscopy in a 15 × 15 × 15-mm3 voxel. Acquisition will be synchronized to the respiratory cycle and triggered at the end of expiration. A water-suppressed lipid spectrum and a lipid-suppressed water spectrum will be acquired in three different locations of the liver to account for liver inhomogeneity. A minimum of three spectra will be acquired for each participant and the total lipid content will be averaged and calculated. In addition, hepatic lipid content will be corrected for transverse relaxation, using the transverse relaxation times of 22 ms for water and 44 ms for lipid.[15] These MRS measurements will take approximately 30 minutes.

Table 1: Study Procedures Schedule

Week	0	16
3-day diet record	√	√
International Physical Activity Questionnaire	√	√
Clinical status and symptoms	√	√
Medication use	√	√
Height	√	
Body weight*	√	√
Comprehensive Metabolic Panel (CMP)	√	
Plasma lipids and lipoproteins	√	√
A1c	√	√
Glucose tolerance testing	√	√
REE	√	√
Postprandial metabolism (TEF)	√	√
Body composition	√	√
IMCL and hepatic lipid (pilot)	√	√

4. INTERVENTION PROCEDURES

4.1. Intervention Diet

The interventions for the Diet and Control Groups are described below.

The Diet Group will be asked to follow a low-fat, vegan diet. According to the Academy of Nutrition and Dietetics, vegan and vegetarian diets meet all nutritional requirements when appropriately planned.[16] The diet consists of whole grains, vegetables, legumes, and fruits, with no restriction on energy intake. Animal products and added oils will be excluded. In choosing grain products and starchy vegetables (e.g., bread, potatoes), participants will be encouraged to select those retaining their natural fiber and having a glycemic index <70, using tables standardized to a value of 100 for glucose. No meals will be provided. Participants will handle their own food preparation and purchases, with guidance from the research team.

The diet derives approximately 10% of energy from fat, approximately 10-15% of energy from protein, and the remainder from complex carbohydrates. The diet will provide approximately 30-40 grams of fiber per day. It is generally adequate in all nutrients except vitamin B_{12}.

Participants will be provided with a commercially available supplement containing 100 micrograms of vitamin B_{12} and asked to take it daily during the study. Should they wish to continue the diet thereafter, they will be counseled to use any standard multivitamin or other reliable source of vitamin B_{12}.

An advantage of studies such as this one, which include volunteers who are not confined to a metabolic ward or otherwise restricted, is that they can readily translate to nonclinical settings. A disadvantage is that they include a degree of uncertainty as to the extent to which participants have adhered to their prescribed diets. While this uncertainty cannot be entirely eliminated, several measures will be taken to maximize dietary adherence, based on the current investigators' published review of factors associated with dietary compliance in clinical trials. Stricter limits on fat intake, frequent monitoring of reported dietary intake, family involvement, group support, and the use of vegetarian diets are associated with a greater degree of dietary change.[17]

Control Group members will be asked to continue their usual diets for the 16-week study period. Those who wish to try the intervention diet will be given instruction in the diet at the study's conclusion.

Both groups: For both groups, alcoholic beverages will be limited to one per day for women, and two for men.

4.2. Dietary Instruction and Group Meetings

Diet Group participants will be asked to attend weekly, one-hour group sessions for support and education. **(Class Curriculum, Appendix 8).** No weekly support or education will be provided to the participants in the Control Group.

All group sessions will be conducted by a registered dietitian, nurse, physician, cooking instructor, or research staff and will include information on nutrition, meal planning, shopping, food preparation techniques, recipes, and everyday dietary challenges, such as dining out and healthful snacking. The classes will also include education on topics such as maintaining a healthy weight, cholesterol, hypertension, diabetes, and other health issues.

For some sessions, participants will be encouraged to bring a spouse, partner, family member, or friend. To facilitate interaction between diet instructors and participants, classes will be conducted in sections of approximately 15 participants.

The curriculum is based on the investigators' prior studies and uses concepts from the Health Belief Model developed by researchers with the Public Health Service and adapted by others.[18] This model describes constructs that predict health-related behaviors and should be considered when planning behavioral change strategies. These include perceived susceptibility, severity, benefits, and barriers, as well as cues to action, and self-efficacy. Our participants are already aware that they are overweight and may benefit from diet changes. Nonetheless, they need help in overcoming barriers and gaining confidence in their ability to implement new dietary habits. We have therefore focused the content of the weekly support group sessions on integrating practical skills (e.g., menu planning, food preparation, dining out, healthful snacking) with their growing understanding of how dietary choices affect health. In order to facilitate individual experience with the prescribed diet, practical skills are presented early, while intellectual understanding of more complex health issues (e.g., how diet affects heart disease risk) is presented later. Each group session includes time for participants to discuss their successes and challenges, and group problem-solving is encouraged.

The study does not seek to separate the effects of the diet from those of regular group support. Rather, group support is a means of facilitating adherence. It should also be emphasized that the goal of this study is not to construct an intervention diet that is isocaloric compared with diets followed by the Control Group participants. Because the intervention diet is low in fat and high in fiber, self-selected energy intake is likely to fall as the diet period begins, and weight loss is likely.

4.3. Exercise and Medication Use

Participants in both groups will be asked to keep their level of physical exercise and use of medications constant and to add no new nutritional supplements to their current medication regimens, except as recommended by their personal physicians.

4.4. Intervention Fidelity and Dietary Adherence

Individual meetings. During the initial individual meal-planning meetings with the Diet Group participants, dietitians will follow a set agenda which will cover the use of vegan

foods, methods for reducing dietary fat, and how to avoid proscribed foods.

Group meetings. To maintain intervention fidelity, the group leaders will follow a set course curriculum, using an agenda for each session and keeping a checklist of major content items to be covered at each meeting.

Dietary Adherence. Each participant will complete diet records at regular intervals using the methods described above. In addition, for the Diet Group, 24-hour multi-pass dietary recalls will be used to assess dietary adherence to assist study personnel in working with individuals who need additional teaching or support. The 24-hour recalls will be performed either by telephone or in person at weeks 3 and 8. These recalls will not be subjected to statistical analysis, but will allow the investigators to check for poor adherence. Such recalls have the advantage that they can be conducted at unscheduled times and over the telephone, and so are not subject to the planning and preparation required for food records.[19] In cases where participants appear to be deviating from the prescribed diet, additional dietary counseling will be provided.

4.5. Participant Retention

Participants' interests for volunteering will be ascertained during screening. Those with reasons for volunteering other than a desire to improve their health or to advance scientific understanding may be rejected. The exclusion criteria also eliminate individuals with a history of unresolved substance abuse, which may influence retention.

Participants will be instructed that attendance at meetings is essential to study participation. The research team will take attendance at each meeting. The research staff will make phone calls to participants who do not attend.

In the weekly meetings, group support will be facilitated through group discussions and encouragement to share successes and difficulties with the prescribed diet. Meeting content will remain varied, including nutrition lectures, health education, cooking demonstrations, and opportunities to taste food. Family members will be invited to certain support group sessions. A voluntary listserv will allow Diet Group members to exchange information, recipes and ideas between meetings. Only participants, study coordinators, and the PI will be allowed to post on the list serve and the content of the list serve will be accessible only by them.

Participants who complete all assessments at weeks 0 and 16 will be paid $100 at completion of their final assessments.

4.6. Biological Specimen Handling Procedures

Samples for the study endpoints will be drawn by LabCorp and will be processed using standard procedures.

5. STATISTICAL PROCEDURES

5.1. Power Analysis

Power Analysis for Overall Study

Sample size will be based on the change in postprandial metabolism (thermic effect of food) previously observed with a plant-based diet, compared with an active dietary control.[3] The current power analysis assumes there will be a single t-test for the comparison of the changes in thermic effect of food observed in the two study groups, with an alpha level of 0.05.

In the prior study, the change at 14 weeks for the thermal effect of food was 4.7 with an SD of 12 in the intervention arm, and 0.3 with an SD of 9.4 in the control arm. Assuming that the true treatment effect is 4.4 kcal/170 min and that the SD of the change will be 12.0 units in the Diet Group and 9.4 units in the Control Group, as previously observed, the sample size required is 96 per arm (192 total) for 80% power, 109 per arm for 85% power, 128 per arm for 90% power.

However, if this magnitude of treatment effect is assumed to be present at the same magnitude of effect at each of the 5 evaluation points used in TEF assessment and assuming that the standard deviation is about 10.85 points for all observations, with 5 observations per participant, correlated at a magnitude of 0.7 with each other, the sample size required is 73 participants per arm (146 total) with 80% power, 83 per arm with 85% power, 98 per arm for 90% power.

Assuming an attrition of 10%, the required sample size is 81 per group, or 162 total for 80% power.

Power Analysis for Intracellular Lipid Substudy

Two studies, cited above, provide a basis for a power analysis for the substudy assessing the role of intramyocellular and hepatic lipid on insulin sensitivity and, ultimately, postprandial metabolism. In the 2012 Yale study, 7 lean, young insulin-resistant individuals whose parents had type 2 diabetes followed a hypocaloric (1200 kcal/d) diet for 9 weeks, leading to an average weight loss of 4.1 ± 0.6 kg. During this intervention period, average intramyocellular lipid fell approximately 30%, from 1.1 ± 0.2% to 0.8 ± 0.1%.[5] In an observational study including 21 individuals following vegan diets and 25 following omnivorous diets, soleus muscle intramyocellular lipid for the vegan participants was found to be 11.7 (6.1–24.6), compared with 16.9 (2.7–44.7) for the omnivorous participants (P = 0.01). The 95% confidence interval for the difference was reported to be -13.2 to -3.3).[7]

Based on the Yale study, assuming a change in IMCL of 0.3 percentage points with a standard deviation of 0.2 and, in the control arm, a mean change of zero with a similar standard deviation, to have 90% power to detect a difference of this magnitude between the two arms would require 11 subjects per arm. Ten per arm would yield 88% power.

Because this is an exploratory substudy and variability in response to the diet is largely unknown, we aim to include 20 participants per arm in the substudy, for a total of 40 participants.

5.2. Data Management

All laboratory samples, reports, questionnaires, and data sheets will be coded with participant identification numbers, rather than names. Laboratory reports will be delivered to the PCRM office at 5100 Wisconsin Avenue, Washington, D.C., where they, along with all other history and data forms, will be maintained in individual participant files in a locked cabinet.

Data will be promptly entered into the data tables at PCRM using Microsoft Excel. Two research staff members will check the tables for accuracy against the original documents. Data tables will be routinely copied onto back-up files and stored for safety on an off-site, passcode-protected, secure server. Data grids will be sent electronically to the biostatistician for analysis. Registered dietitians will be provided with information on usual ranges for nutrients or intakes of interest and asked to check their original data and analysis for errors if they fall outside of these ranges.

5.3. Statistical Analysis

Descriptive statistics for all demographic variables and clinical measures will be calculated for each group. To determine if there are statistically significant differences between the 2 groups at baseline, t-tests will be calculated for continuous measures and chi squares will be calculated for categorical measures. Regardless of any differences, baseline values for key outcome variables will be included as covariates in the main assessments of the effect of diet in the multivariate analysis of covariance. An alpha of 0.05 will be used for all statistical tests.

For nutrient intake and physical measures, descriptive statistics (means, standard deviations, tests for normality) will be calculated. If data are normally distributed, parametric tests for significant effects will be used; for non-normally distributed variables, non-parametric tests will be used.

The initial test of the hypotheses will be examined by performing t-tests for independent samples on the difference score denoting the change from baseline to the reporting period. For missing data in a reporting period, values from the previous period will be brought forward. For body weight, drop-outs will be considered to have returned to baseline weights.

5.4. Assessment of Diet Adherence. Diet-and-supplement group participants will be described as adherent or non-adherent based on whether they met the following criteria: absence of proscribed foods reported on 24-hour recalls and diet records, saturated fat $<5\%$ and total fat $<25\%$ of energy, and average daily cholesterol intake <50 mg on 3-day dietary records.

For drop-out rates, we will determine if there are between-group differences, using chi-square.

5.5. Assessment of Medication use. Any changes to lipid-lowering medications will be classified as a net increase, net decrease, or mixed (changes in opposing directions for 2 or more medications). Using chi-square, we will determine whether there are differences in medication changes between the 2 groups.

6. TIME LINE AND PARTICIPANT FLOW

6.1. Time Line

The study will be conducted in six cohorts, each including 40 participants, over a 3-year period.

For the first cohort, recruitment will take place October 2016 – January 2017. The intervention, including weekly meetings, will take place between February and May 2017 for a total of 16 weeks. For the second cohort, recruitment will take place in December 2016 and January, 2017. The intervention, including weekly meetings, will take place between March and July 2017. Similar time frames will apply in the two subsequent years.

6.2. Participant Flow Based on Power Analysis

In order to accommodate this number of participants, baseline metabolic assessments will occur as follows for the first cohort:

January 6: 2 participants
January 9-13: 10 participants
January 17-20: 8 participants
January 23-27: 10 participants
January 30-Feb 3: 10 participants

The MR spectroscopy studies will be limited to 40 participants (20 per study arm) total. These evaluations will be scheduled during the above scheduled days.

The Diet Group sessions for the first cohort will be held for 16 weeks, from February 1 through May 17, 2017.

For the first cohort in 2017, the 16-week metabolic assessments will occur as follows:

May 18-19: 4 participants
May 22-26: 10 participants
May 30-June 2: 8 participants

June 5-9: 10 participants
June 12-15: 8 participants

For the second cohort, the assessment and intervention dates will be approximately one month later than those for the first cohort.

7. PROTECTION OF HUMAN RESEARCH PARTICIPANTS

7.1. Risks to the Subjects

Sources of Materials: Participants will be asked to complete questionnaires, provide blood samples, and have several physical assessments.

Human Subjects Involvement and Characteristics: The proposed research will include participants at least 18 years of age.

Potential Risks: Participation in the study entails the following risks:

1. Blood draws can cause transient pain, occasionally cause bruising, and may cause bleeding.

2. A well-planned vegan diet provides all the nutrients people need except for vitamin B12. People with a vitamin B12 deficiency may suffer from anemia and neurologic damage.

3. Loss of confidential information.

7.2. Adequacy of Protection against Risks

Recruitment and Informed Consent: Participants will be informed of the study's goals and procedures and review the inclusion and exclusion criteria. Volunteers who appear to meet the criteria for participation will be invited to a group or individual interview with the principal investigator and the study coordinator, who will explain the study in detail, answer questions, and provide a written consent form, as approved by the IRB. Participants will have the opportunity to ask any questions individually in a private setting and may take as much time as they would like to review the informed consent document. The consent form will be signed by the volunteer participant and study coordinator. The principal investigator will certify that the research study has been explained to the volunteer, including the purpose, procedures, possible risks, and potential benefits associated with participation and that any questions have been answered to the volunteer's satisfaction.

Participants' personal physicians will be notified of their involvement in the study and that the investigators will not manage any aspects of their medical care.

To maintain confidentiality, all laboratory specimens, questionnaires, forms, and data sheets will identify participants by their assigned numbers only. Data and safety monitoring are described below.

Phlebotomy risks. All blood draws will be carried out by experienced phlebotomists at Quest Diagnostics.

Vitamin B12 deficiency. All Diet Group participants will be given a supply of vitamin B12, 100 micrograms, and will be asked to take it daily. Participants will also be counseled to continue B12 supplementation if they plan to continue following a vegan diet.

Loss of confidential information. We will make every effort to keep all research records private to the extent allowed by law. We will use an identification number on forms, instead of identifiable information. All study documents will be kept in locked filing cabinets and in password protected electronic files at PCRM's office. Information we learn from this study may be shared at scientific or medical meetings and may be published, but participants will not be personally identified.

Our protocol also includes the following safeguards:

1. All participants will remain under the care of their personal healthcare providers.

2. All participants will continue on the medications they were using at study entry, unless modified by their personal physician(s).

We therefore believe that the risks to participants in a dietary intervention trial are minimal, while the scientific and public health merit of such an investigation is high. By studying the benefits of a dietary intervention, we hope to obtain valuable research data.

Confidentiality. To maintain confidentiality, all laboratory specimens, questionnaires, forms, and data sheets will identify participants by their assigned numbers only. Data and safety monitoring are described below.

7.3. Potential Benefits of the Proposed Research to the Participants and Others

Given that, over the long run, excess body weight contributes to morbidity and mortality, the dietary instruction and consistent support provided may be of substantial benefits for the Diet Group. The Control Group will be given detailed information on how to follow the intervention diet at the study's conclusion.

7.4. Importance of the Knowledge to Be Gained

Weight problems are extremely common, and gaps remain in our understanding of how intervention diets work. This study is founded on clear theoretical constructs and compelling previous data on both the efficacy and acceptability of the experimental

intervention, as well as preliminary findings on its mechanisms of action. It investigates what may be a major advance in the understanding of the role of diet in weight control. The risks to participants are small, and the potential benefits are significant.

7.5. Assessment and Reporting of Adverse Events

An adverse event is any adverse physical or clinical change experienced by a participant. This includes the onset of new symptoms and the exacerbation of pre-existing conditions. In order to avoid bias in eliciting reports of adverse events, participants will be asked, during assessments at the end of each study period, "Have you had any new symptoms, injuries, illness or side effects or worsening of pre-existing conditions?" at each visit.

All adverse events will be recorded in the participant's record and on the IRB continuing review form. The severity of the adverse event will be assessed, and actions/outcomes (e.g., hospitalization, discontinuation of therapy, etc.) will also be recorded.

Any actions taken and follow-up results will also be recorded on the appropriate page of the IRB continuing review form, as well as in the participant's record. Follow-up laboratory results will be filed with the participant's record. All adverse events occurring at a site will be reported by the investigator to the IRB according to the Data and Safety Monitoring Plan, described below.

The following definitions will be used:

Minimally serious:	Awareness of sign, symptom, or event, but easily tolerated.
Somewhat Serious:	Discomfort enough to cause interference with usual activity and may warrant investigation.
Very Serious:	Incapacitating, with inability to do usual activities, or significantly affects clinical status, and warrants intervention.
Life-threatening:	Immediate risk of death.

The research team will also assess the relationship of any adverse event to the study intervention, based on available information, using the following guidelines:

0 = Unlikely	No temporal association, or the cause of the event has been identified, or the study interventions cannot be implicated.
1 = Possibly	Temporal association, but other etiologies are likely the cause; however, involvement of the study interventions cannot be excluded.

2 = Probably	Temporal association or other etiologies are possible, but unlikely.

7.6. Serious Adverse Events (SAEs)

All serious adverse events, whether or not deemed intervention-related or expected, will be reported by telephone to the Safety Officer within 24 hours (one working day) of the time they become known. A written report will follow as soon as possible, including a full description of the event and any sequelae. This includes serious events that occur any time after the inclusion of the patient in the study until completion of the last visit. A serious adverse event report will also be sent via fax to the IRB chair.

A serious adverse event is any event that falls in any of the following categories:

- Fatal
- Life-threatening (the patient was at immediate risk of death from the AE as it occurred)
- Significantly or permanently disabling
- Requires hospitalization or prolongs hospitalization

Important medical events that may not result in death, be life-threatening, or require hospitalization may be considered serious adverse events when, upon appropriate medical judgment, they may jeopardize the patient and may require medical or surgical intervention to prevent one of the outcomes listed in the definition. The death of any patient during the study, regardless of the cause, will be reported within 24 hours by telephone to the Safety Officer and IRB. A full written report will follow as soon as possible. If an autopsy is performed, a copy will be provided to the Safety Officer and IRB.

Reports of all serious adverse events, including deaths, will be communicated to the IRB in accordance with local laws and regulations.

7.7. Action plan if a subject becomes severely depressed or suicidal during the course of the study

Participants with a history of severe mental illness (with current unstable status), such as severe depression or suicidality, will not be enrolled in the study as indicated in the exclusion criteria. If a participant becomes severely depressed during the course of the study, he or she will be referred to see his or her primary care physician or psychiatrist and to seek medical care. The event will be recorded and reported to the PI and Safety Officer immediately. The PI will notify IRB within 24 hours of recognition of the event by study personnel. All non-serious events will be reported and reviewed by the PI within one week. Study personnel will inform the primary care physician of all events occurring in his/her patients within 48 hours of recognition of the event.

If a participant becomes suicidal during the course of the study, he or she will be instructed to call 911 and seek emergency medical care. The incident will be recorded and reported to the PI and Safety Officer immediately. The PI will notify the IRB within 24 hours of recognition of the event by study personnel. All non-serious events will be reported and reviewed by the PI within one week. Study personnel will inform the primary care physician of all events occurring in his/her patients within 48 hours of recognition of the event.

7.8. Data and Safety Monitoring Plan

Data and Safety Monitoring functions will be performed by the principal investigator (PI, Neal D. Barnard, M.D.), study coordinator (Francesca Valente), study statistician, and a Safety Officer who is a physician who is not part of the research staff and has no role in care of the participants. The Safety Officer will have no scientific, financial, or other conflict of interest related to the trial. Prior to the study onset, the study statistician and Safety Officer will review the research protocol, informed consent documents, and plans for data and safety monitoring.

During the recruitment phase, the study coordinator and PI will review enrollment weekly. At monthly intervals, they will produce a report describing the study's progress, including accrual, demographics, thoroughness of baseline data, subject status (reporting concurrent illnesses, withdrawal of consent, or loss to follow-up), and adherence to participation criteria, informed consent procedures, and the study protocol. The reports will be submitted to the study statistician and Safety Officer.

Prior to participant randomization, a full history of each participant's pre-existing symptoms and medical problems will be recorded. During each monitoring visit, study participants will be asked if any medical symptom, problem, or event has occurred or if there has been any change in pre-existing symptoms.

All serious events (hospitalization, serious illness, or disability) will be recorded and reported to the PI and Safety Officer. The PI will notify the IRB within 24 hours of recognition of the event by study personnel. All non-serious events will be reported and reviewed by the PI within one week. Study personnel will inform the primary care physician of all events occurring in his/her patients within 48 hours of recognition of the event.

At monthly intervals, the study coordinator and PI will prepare and submit to the study statistician and Safety Officer a report covering each of the following areas: (1) performance (including adherence to the study protocol and maintenance of data integrity and confidentiality), (2) safety (including abnormal laboratory values, adverse events, serious adverse events, deaths, and disease- or treatment-specific events), and (3) treatment effects, including medication changes.

The study statistician and Safety Officer will review each safety report within one week of receipt. The study statistician will review these reports to assess whether event rates

are of statistical concern and, if so, will alert the Safety Officer, the PI, and the IRB. The study statistician and Safety Officer will also consider factors external to the study, e.g., new scientific developments, that may affect the safety of participants or the conduct of the trial.

The Safety Officer will make recommendations as necessary to the PI. If the Safety Officer recommends a study change for patient safety or for ethical reasons, or if the study is closed early due to slow accrual, the PI will be responsible for implementing the recommendations as expeditiously as possible. If the PI does not concur with any recommendation of the Safety Officer, both will be responsible for reaching a mutually acceptable decision.

7.9. Stopping Rules

At the conclusion of the 16-week intervention period for the first cohort, the study statistician will prepare a report on clinical changes and adverse events for presentation to the PI and the Safety Officer. If evidence available at that point clearly shows either (1) an effect of the intervention diet on postprandial metabolism or (2) harm associated with the intervention diet, the Safety Officer may recommend early termination of the study.

8. INCLUSION OF WOMEN, MINORITIES, AND CHILDREN

8.1. Inclusion of Women

The participation criteria, cited above, include both men and women. Recruitment procedures are expected to yield roughly equal numbers of men and women.

8.2. Inclusion of Minorities

The U.S. Census Bureau reports both race and ethnicity, the latter term used primarily to denote self-identification as Hispanic or non-Hispanic. According to the 2015 Census Bureau, races were represented in Washington, DC, as follows: 48.3% black, 44.1% white, 4.2% Asian, 0.6% American Indian/Native American, 0.2% Native Hawaiian or other Pacific Islander; 2.7% 2 or more races. In addition, 10.6% of the population identified themselves as Hispanic.[20] In our prior studies, the respondent populations have been demographically diverse, reflecting the profile of the greater Washington, D.C area.

8.3. Inclusion of Children.

Persons less than 18 years of age will not be included in the study because they have insufficient control over the dietary choices that are essential to meaningful participation.

9. BRIEF STATEMENT OF ANTICIPATED OUTCOMES

This study aims to test hypotheses that are potentially important for individual and public health. It will improve our understanding of the treatment of weight problems and will also have practical implications for reducing the medical, personal, and economic costs associated with obesity. Anticipated outcomes for Diet Group participants include beneficial changes in body weight, insulin sensitivity, and serum lipid concentrations, all of which are also possible for the Control Group participants who choose to take advantage of instruction in the intervention diet at the study's conclusion.

LITERATURE CITED

[1] Tonstad, S, Butler T, Yan R, Fraser GE.Type of vegetarian diet, body weight and prevalence of type 2 diabetes. Diabetes Care. 2009;32:791-6.

[2] Barnard ND, Levin SM, Yokoyama Y. A systematic review and meta-analysis of changes in body weight in clinical trials of vegetarian diets. J Acad Nutr Diet. 2015 Jun;115(6):954-69.

[3] Barnard ND, Scialli AR, Turner-McGrievy G, Lanou AJ, Glass J. The effects of a low-fat, plant-based dietary intervention on body weight, metabolism, and insulin sensitivity. Am J Med 2005;118:991-997.

[4] Shulman GI. Ectopic fat in insulin resistance, dyslipidemia, and cardiometabolic disease. N Engl J Med 2014;371:1131-1141

[5] Petersen KF, Dufour S, Morino K, Yoo PS, Cline GW, Shulman GL. Reversal of muscle insulin resistance by weight reduction in young, lean, insulin-resistant offspring of parents with type 2 diabetes. PNAS. 2012;109:8236-40.

[6] Sparks LM, Xie H, Koza RA, et al. A high-fat diet coordinately downregulates genes required for mitochondrial oxidative phosphorylation in skeletal muscle. Diabetes. 2005;54:1926–33.

[7] Goff LM, Bell JD, So PW, Dornhorst A, Frost GS. Veganism and its relationship with insulin resistance and intramyocellular lipid. Eur J Clin Nutr. 2005;59:291–298.

[8] Barnard N, Scherwitz L, Ornish D. Adherence and acceptability of a lowfat vegetarian diet among patients with cardiac disease. *J Cardiopulmonary Rehabil* 1992;**12**:423-31.

[9] Barnard N, Scialli A, Bertron P, Hurlock D, Edmonds K. Acceptability of a therapeutic low-fat, vegan diet in premenopausal women. *J Nutr Educ* 2000;**32**:314-9.

[10] Barnard ND, Scialli AR, Turner-McGrievy G, Lanou AJ. Acceptability of a low-fat vegan diet compares favorably to a step II diet in a randomized, controlled trial. *Journal of cardiopulmonary rehabilitation* 2004;**24**(4):229-35.

[11] Barnard ND, Gloede L, Cohen J, et al. A low-fat vegan diet elicits greater macronutrient changes, but is comparable in adherence and acceptability, compared with a more conventional diabetes diet among individuals with type 2 diabetes. *J Am Diet Assoc* 2009;**109**(2):263-72.

[12] Craig CL, Marshall AL, Sjostrom M, et al. International physical activity questionnaire: 12-country reliability and validity. *Medicine and science in sports and exercise* 2003;**35**(8):1381-95.

[13] Petersen KF, et al. (2006) Increased prevalence of insulin resistance and nonalcoholic fatty liver disease in Asian-Indian men. Proc Natl Acad Sci USA 103:18273–18277.

[14] Gruetter R (1993) Automatic, localized in vivo adjustment of all first- and second-order shim coils. Magn Reson Med 29:804–811.

[15] Rabøl R, Petersen KF, Dufour S, Flannery C, Shulman GI (2011) Reversal of muscle insulin resistance with exercise reduces postprandial hepatic de novo lipogenesis in insulin resistant individuals. Proc Natl Acad Sci USA 108:13705–13709.

[16] Position of the American Dietetic Association and Dietitians of Canada: Vegetarian diets. *J Am Diet Assoc* 2003;**103**(6):748-65.

[17] Barnard N, Akhtar A, Nicholson A. Factors that facilitate dietary change. *Arch Fam Med* 1995;**4**:153-8.

[18] Becker M. The health belief model and personal health behavior. *Health Education Monographs* 1974;**2**:324-473.

[19] Buzzard I, Faucett C, Jeffery R, et al. Monitoring dietary change in a low-fat diet intervention study: advantages of using 24-hour dietary recalls vs food records. *J Am Diet Assoc* 1996;**96**:574.

[20] U.S. Census Bureau. Quick Facts. District of Columbia. Internet: http://www.census.gov/quickfacts/table/RHI125215/11, accessed August 22, 2016.
