# Supplementary material for: Effect of a Low-Fat Vegan Diet on Body Weight, Insulin Sensitivity, Postprandial Metabolism, and Intramyocellular and Hepatocellular Lipid Levels in Overweight Adults: A Randomized Clinical Trial
Source: JAMA Netw Open. 2020 Nov 30;3(11):e2025454. doi: 10.1001/jamanetworkopen.2020.25454 (PMC7705596; doi:10.1001/jamanetworkopen.2020.25454)
Supplement: Supplement 2. — eTable 1. Baseline characteristics of the study population eTable 2. Baseline characteristics of the study population, comparing study completers and drop-outs eTable 3. Baseline characteristics of the study population, comparing the subsample undergoing magnetic resonance spectroscopy (MRS) with the rest of the study population eTable 4. Treatment effects for the main outcomes, adjusted for age and race eFigure 1. Changes in the thermic effect of food, liver fat, and intramyocellular lipids after adjustment for race and age eFigure 2. Changes in liver fat and intramyocellular lipids after adjustment for baseline BMI eFigure 3. Linear regression model for changes in energy and body weight and postprandial energy expenditure and body weight [file jamanetwopen-e2025454-s002.pdf]

Supplemental Online Content

Kahleova H, Petersen KF, Shulman GI, et al. Effect of a low-fat vegan diet on body weight, insulin sensitivity, postprandial metabolism, and intramyocellular and hepatocellular lipid levels in overweight adults: a randomized clinical trial. *JAMA Netw Open*. 2020;3(11):e2025454. doi:10.1001/jamanetworkopen.2020.25454

eTable 1. Baseline characteristics of the study population

eTable 2. Baseline characteristics of the study population, comparing study completers and drop-outs

eTable 3. Baseline characteristics of the study population, comparing the subsample undergoing magnetic resonance spectroscopy (MRS) with the rest of the study population

eTable 4. Treatment effects for the main outcomes, adjusted for age and race

eFigure 1. Changes in the thermic effect of food, liver fat, and intramyocellular lipids after adjustment for race and age

eFigure 2. Changes in liver fat and intramyocellular lipids after adjustment for baseline BMI

eFigure 3. Linear regression model for changes in energy and body weight and postprandial energy expenditure and body weight

This supplemental material has been provided by the authors to give readers additional information about their work.

© 2020 Kahleova H et al. *JAMA Network Open.*

Characteristic	Intervention group (n=122)	Control group (n=122)	P Value
**Age (years, SD)**	53 (±10)	57 (±13)	0.01
**Sex (number, %)**			0.85
Female	105 (86.0)	106 (86.8)	
Male	17 (14.0)	16 (13.1)	
**Race, (number, %)**			0.06
White	57 (46.7)	60 (49.2)	
Black	60 (49.2)	53 (43.4)	
Asian, Pacific Islander	1 (0.8)	7 (5.7)	
American Indian, Eskimo, Aleut	2 (1.6)	0 (0.0)	
Did not disclose	2 (1.6)	2 (1.6)	
**Ethnicity, (number, %)**			0.75
Non-Hispanic	97 (79.5)	101 (82.8)	
Hispanic	8 (6.6)	7 (5.7)	
Did not disclose	17 (13.9)	14 (11.5)	
**Marital status, (number, %)**			0.87
Not married	66 (54.1)	61 (50.0)	
Married	55 (45.1)	53 (43.4)	
Did not disclose	1 (0.8)	8 (6.6)	
**Education, (number, %)**			0.15
High school	8 (6.6)	11 (9.0)	
Associates	35 (28.7)	43 (35.2)	
College	72 (59.0)	63 (51.6)	
Graduate degree	7 (5.7)	5 (4.1)	
**Occupation, (number, %)**			0.23
Service occupation	27 (22.1)	15 (12.3)	
Technical, sales, administrative	34 (27.9)	32 (26.2)	
Professional or managerial	33 (27.0)	39 (32.0)	
Retired	16 (13.1)	24 (19.7)	
Other →	12 (9.8)	12 (9.8)	
**Medications, (number, %)**			
Lipid-lowering therapy (%)	22 (18.0)	21 (17.2)	0.87
Antihypertensive therapy (%)	33 (27.0)	31 (25.4)	0.77
Thyroid medications (%)	16 (13.1)	12 (9.8)	0.42
Total physical activity (number, SD)	2719 (±4701)	2863 (±3085)	0.80
**Dietary Intake (number, SD)**			
Caloric intake (kcal/day)	1834 (±574)	1793 (±628)	0.61

© 2020 Kahleova H et al. *JAMA Network Open.*

Total fiber intake (g/day)	24.1 (±10.6)	23.9 (±10.1)	0.89
Total cholesterol intake (mg/day)	238.6 (±143.9)	244.5 (±169.3)	0.78
Total saturated fatty acid intake (g/day,)	23.6 (±12.0)	22.9 (±12.2)	0.65
Monounsaturated fatty acids (g/day)	27.2 (±10.4)	27.9 (±13.9)	0.67
Polyunsaturated fatty acids (g/day)	18.4 (±8.2)	19.1 (±10.5)	0.59
**Anthropometric variables, (number, SD)**			
Body weight (kg)	93.6 (±13.8)	92.7 (±13.7)	0.62
BMI (kg/m^2)	33.3 (±3.8)	33.6 (±3.7)	0.57
Lean mass (kg)	50.5 (±7.9)	49.5 (±8.1)	0.35
Fat mass (kg)	40.6 (±9.2)	40.9 (±9.6)	0.76
Visceral fat volume (cm^3)	1459 (±944.2)	1517 (±907.0)	0.64
**Laboratory variables (number, SD)**			
Total cholesterol (mmol/l)	5.2 (±1.1)	5.0 (±1.3)	0.11
HDL-cholesterol (mmol/l)	1.6 (±0.4)	1.7 (±0.9)	0.16
LDL-cholesterol (mmol/l)	3.0 (±0.9)	2.9 (±1.1)	0.16
Triglycerides (mmol/l)	1.2 (±0.5)	1.3 (±0.6)	0.10
Fasting plasma glucose (mmol/l)	5.2 (±0.2)	5.0 (±0.3)	0.18
Fasting plasma insulin (pmol/l)	91.2 (±59.6)	78.9 (±51.1)	0.12
HbA1c (DCCT, %)	5.6 (±0.4)	5.7 (±0.4)	0.30
**Insulin sensitivity /resistance (number, SD)**			
PREDIM (mg/min/kg)	4.1 (±1.3)	4.4 (±1.5)	0.11
HOMA-IR (dimensionless)	3.2 (±2.2)	2.7 (±1.9)	0.17

eTable 1. Baseline characteristics of the study population. Data are means ± SD (standard deviation), or number (%). P-values refer to t-tests for continuous variables and χ^2 (chi-squared) or Fisher's exact test for categorical variables. The P-value calculated for ethnicity distribution is for the comparison between Hispanic vs. non-Hispanic categories (and all other comparisons also exclude datapoints that were not available).

© 2020 Kahleova H et al. *JAMA Network Open.*

Characteristic	Drop-outs (n=22)	Study completers (n=222)	P Value
**Age (years, SD)**	57.8 (±12.5)	54.4 (±11.6)	0.20
**Sex (number, %)**			
Female	19 (86.4)	192 (86.5)	1.0
Male	3 (13.6)	30 (13.5)	
**Race, (number, %)**			0.30
White	7 (31.8)	110 (49.6)	
Black	14 (63.6)	99 (44.6)	
Asian, Pacific Islander	1 (4.5)	7 (3.2)	
American Indian, Eskimo, Aleut	0 (0)	2 (0.9)	
Did not disclose	0 (0)	4 (1.8)	
**Ethnicity, (number, %)**			1.0
Non-Hispanic	18 (81.8)	180 (81.1)	
Hispanic	1 (4.5)	14 (6.3)	
Did not disclose	3 (13.6)	28 (12.6)	
**Marital status, (number, %)**			0.09
Not married	15 (68.2)	112 (50.5)	
Married	6 (27.3)	102 (45.9)	
Did not disclose	1 (4.5)	8 (3.6)	
**Education, (number, %)**			0.58
High school	3 (13.6)	16 (7.2)	
Associates	0 (0)	1 (0.5)	

© 2020 Kahleova H et al. *JAMA Network Open.*

College	8 (36.4)	81 (36.5)	
Graduate degree	11 (50.0)	123 (55.4)	
Did not disclose	0 (0)	1 (0.5)	
**Occupation, (number, %)**			0.49
Service occupation	4 (18.2)	38 (17.1)	
Technical, sales, administrative	7 (31.8)	59 (26.6)	
Professional or managerial	3 (13.6)	69 (31.1)	
Retired	5 (22.7)	35 (15.8)	
Other	2 (9.1)	21 (9.5)	
Did not disclose	1 (4.5)	0 (0)	
**Medications, (number, %)**			
Lipid-lowering therapy (%)	6 (27.3)	39 (17.6)	0.24
Antihypertensive therapy (%)	6 (27.3)	59 (26.6)	0.84
Thyroid medications (%)	1 (4.5)	27 (12.2)	0.48
**Total physical activity (number, SD)**	2863 (±3144)	2780 (±4006)	0.93
**Dietary Intake (number, SD)**			
Caloric intake (kcal/day)	1589 (±442)	1812 (±599)	0.13
Total fiber intake (g/day)	20.3 (±6.8)	23.9 (±10.3)	0.16
Total cholesterol intake (mg/day)	197 (±126)	242 (±155)	0.25
Total saturated fatty acid intake (g/day,)	18.8 (±10.5)	23.2 (±12.1)	0.14
Monounsaturated fatty acids (g/day)	23.8 (±11.0)	27.5 (±12.1)	0.22

© 2020 Kahleova H et al. *JAMA Network Open.*

Polyunsaturated fatty acids (g/day)	14.9 (±7.0)	18.6 (±9.3)	0.11
**Anthropometric variables, (number, SD)**			
Body weight (kg)	92.3 (±9.02	93.0 (±13.7)	0.74
BMI (kg/m^2)	33.0 (±2.7)	33.4 (±3.7)	0.56
Lean mass (kg)	50.0 (±7.5)	49.9 (±8.0)	0.98
Fat mass (kg)	40.5 (±4.9)	40.7 (±9.3)	0.88
Visceral fat volume (cm^3)	1355 (±575)	1496 (±914)	0.34
**Laboratory variables (number, SD)**			
Total cholesterol (mmol/l)	5.2 (±0.9)	5.2 (±1.1)	0.78
HDL-cholesterol (mmol/l)	1.5 (±0.4)	1.6 (±0.4)	0.55
LDL-cholesterol (mmol/l)	3.1 (±0.7)	3.1 (±0.9)	0.99
Triglycerides (mmol/l)	1.2 (±0.6)	1.2 (±0.5)	0.92
Fasting plasma glucose (mmol/l)	5.5 (±0.9)	5.4 (±0.6)	0.61
Fasting plasma insulin (pmol/l)	73.2 (±31.9)	78.8 (±62.2)	0.52
HbA1c (DCCT, %)	5.7 (±0.5)	5.7 (±0.4)	0.86
**Insulin sensitivity /resistance (number, SD)**			
PREDIM (mg/min/kg)	3.9 (±1.1)	4.2 (±1.4)	0.58
HOMA-IR (dimensionless)	3.2 (±1.8)	3.0 (±2.0)	0.78

© 2020 Kahleova H et al. *JAMA Network Open.*

eTable 2. Baseline characteristics of the study population, comparing study completers and drop-outs. Data are means ± SD (standard deviation), or number (%). P-values refer to t-tests for continuous variables and χ^2 (chi-squared) or Fisher's exact test for categorical variables. The P-value calculated for ethnicity distribution is for the comparison between Hispanic vs. non-Hispanic categories (and all other comparisons also exclude datapoints that were not available).

**Characteristic**	**MRS YES (n=44)**	**MRS NO (n=200)**	**P-value**
**Age (years, SD)**	55.8 (±11.1)	54.5 (±11.8)	0.52
**Sex (number, %)**			
Female	35 (79.5)	176 (88.0)	0.14
Male	9 (20.5)	24 (12.0)	
**Race, (number, %)**			0.07
White	25 (56.8)	92 (46.0)	
Black	15 (34.1)	98 (49.0)	
Asian, Pacific Islander	3 (6.8)	5 (2.5)	
American Indian, Eskimo, Aleut	1 (2.3)	1 (0.5)	
Did not disclose	0	4 (2.0)	
**Ethnicity, (number, %)**			0.50
Non-Hispanic	37 (84.1)	161 (80.5)	
Hispanic	4 (9.1)	11 (5.5)	
Did not disclose	3 (6.8)	28 (14.0)	
**Marital status, (number, %)**			0.81
Not married	22 (50.0)	105 (52.5)	
Married	20 (45.5)	88 (44.0)	

© 2020 Kahleova H et al. *JAMA Network Open.*

Did not disclose	2 (4.5)	7 (3.5)	
**Education, (number, %)**			
High school	1 (2.3)	18 (9.0)	
Associates	0 (0)	1 (0.5)	
College	24 (54.5)	65 (32.5)	
Graduate degree	19 (43.2)	115 (57.5)	
Did not disclose	0 (0)	1 (0.5)	
**Occupation, (number, %)**			0.30
Service occupation	5 (11.4)	37 (18.5)	
Technical, sales, administrative	17 (38.6)	49 (24.5)	
Professional or managerial	12 (27.3)	60 (30.0)	
Retired	5 (11.4)	35 (17.5)	
Other	5 (11.4)	18 (9.0)	
Did not disclose	0 (0)	1 (0.5)	
**Medications, (number, %)**			
Lipid-lowering therapy (%)	7 (15.9)	38 (19.0)	0.62
Antihypertensive therapy (%)	10 (22.7)	55 (27.5)	0.51
Thyroid medications (%)	4 (9.1)	24 (12.0)	0.58
**Total physical activity (number, SD)**	2711 (±3222)	2804 (±4080)	0.89
**Dietary Intake (number, SD)**			
Caloric intake (kcal/day)	1886 (±629)	1776 (±582)	0.27
Total fiber intake (g/day)	25.7 (±11.9)	23.2 (±9.7)	0.14

© 2020 Kahleova H et al. *JAMA Network Open.*

Total cholesterol intake (mg/day)	255.8 (±188)	234.6 (±145.0)	0.49
Total saturated fatty acid intake (g/day,)	24.3 (±12.0)	22.6 (±12.0)	0.39
Monounsaturated fatty acids (g/day)	29.2 (±14.9)	26.8 (±11.3)	0.34
Polyunsaturated fatty acids (g/day)	19.8 (±12.4)	18.0 (±8.3)	0.36
**Anthropometric variables, (number, SD)**			
Body weight (kg)	89.8 (±11.7)	93.6 (±13.7)	0.08
BMI (kg/m^2)	32.1 (±2.3)	33.7 (±3.8)	<0.001
Lean mass (kg)	50.5 (±8.5)	49.8 (±7.8)	0.60
Fat mass (kg)	37.3 (±7.0)	41.4 (±9.3)	0.002
Visceral fat volume (cm^3)	1559 (±975)	1468 (±874)	0.55
**Laboratory variables (number, SD)**			
Total cholesterol (mmol/l)	5.4 (±1.2)	5.2 (±1.0)	0.27
HDL-cholesterol (mmol/l)	1.6 (±0.6)	1.6 (±0.4)	0.41
LDL-cholesterol (mmol/l)	3.2 (±0.9)	3.1 (±0.9)	0.42
Triglycerides (mmol/l)	1.2 (±0.6)	1.2 (±0.5)	0.91
Fasting plasma glucose (mmol/l)	5.5 (±0.7)	5.4 (±0.6)	0.35
Fasting plasma insulin (pmol/l)	66.4 (±53.3)	81.1 (±72.0)	0.07
HbA1c (DCCT, %)	5.9 (±0.5)	5.6 (±0.4)	<0.001
**Insulin sensitivity /resistance (number, SD)**			

© 2020 Kahleova H et al. *JAMA Network Open.*

PREDIM (mg/min/kg)	4.2 (±1.3)	4.2 (±1.4)	0.78
HOMA-IR (dimensionless)	2.8 (±1.9)	3.0 (±2.0)	0.58

eTable 3. Baseline characteristics of the study population, comparing the subsample undergoing magnetic resonance spectroscopy (MRS) with the rest of the study population. Data are means ± SD (standard deviation), or number (%). P-values refer to t-tests for continuous variables and χ^2 (chi-squared) or Fisher's exact test for categorical variables. The P-value calculated for ethnicity distribution is for the comparison between Hispanic vs. non-Hispanic categories (and all other comparisons also exclude datapoints that were not available).

Outcomes	Treatment effect (adj.)	P-value
Weight (kg)	-6.1 (-7.0 to -5.2)	<.001
BMI (kg/m^2)	-2.4 (-3.6 to -1.2)	<.001
Fat mass (kg)	-4.1 (-4.7 to -3.5)	<.001
Lean mass (kg)	-1.5 (-2.0 to -1.1)	<.001
VAT volume (cm^3)	-217.4 (-316.2 to -118.7)	<.001

© 2020 Kahleova H et al. *JAMA Network Open.*

Hepatocellular lipids (%)	-1.1 (-2.3 to -0.04)	<.001
Intramyocellular lipids (%)	-0.1 (-0.5 to +0.2)	0.02
PREDIM	+0.8 (+0.5 to +1.1)	<.001
HOMA	-1.2 (-2.2 to -0.2)	0.01

eTable 4. Treatment effects for the main outcomes, adjusted for age and race.

© 2020 Kahleova H et al. *JAMA Network Open.*

A: Thermic effect of food in White participants

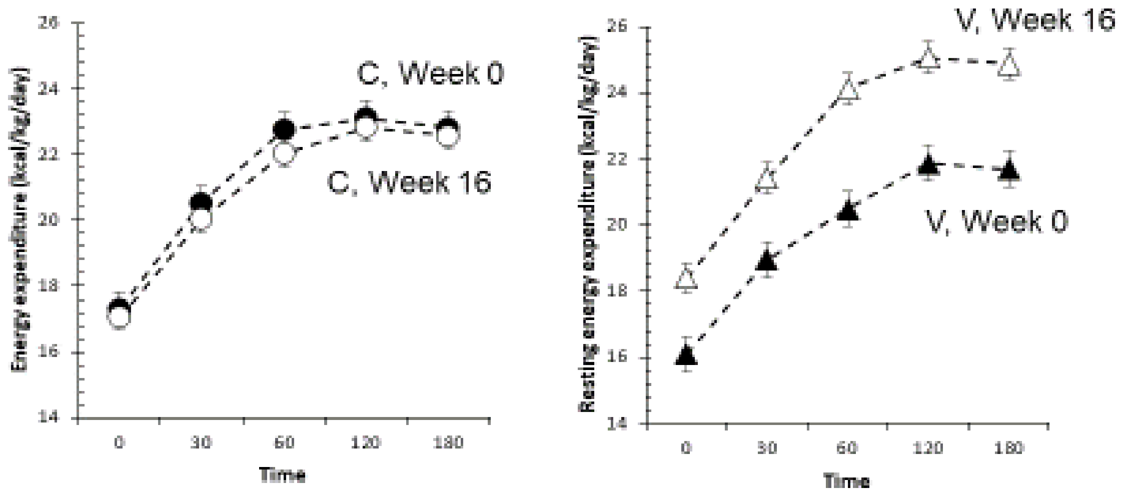

B: Thermic effect of food in Black participants

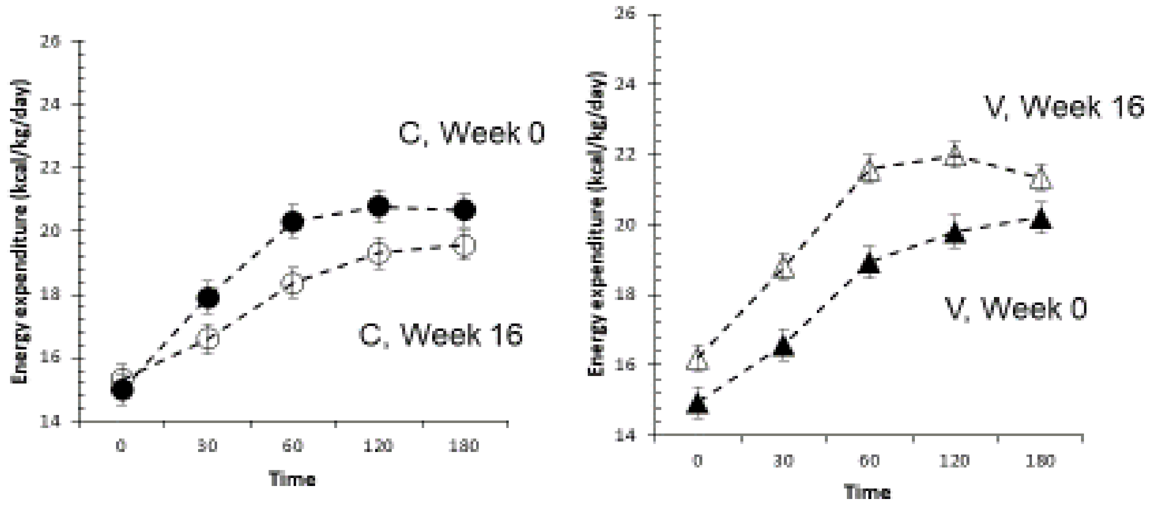

© 2020 Kahleova H et al. *JAMA Network Open.*

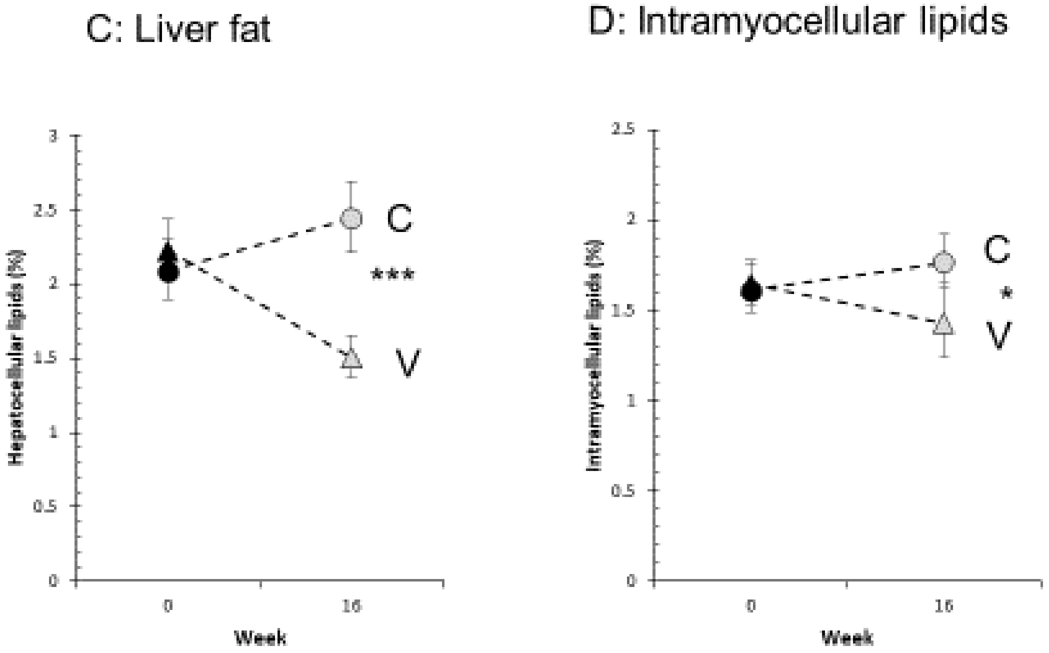

Supplemental Figure 1. Changes in the thermic effect of food (A and B), liver fat (C), and intramyocellular lipids (D) after adjustment for race and age.
Circles are used for the control group (C) and triangles for the vegan group (V).
Full symbols are used for week 0 and empty symbols for week 16.
A: Thermic effect of food in White participants: Group: F=1.3, p=0.26; Age: F=113.4, p<0.001; Week: F=13.5, p<0.001; Time: F=208.1, p<0.001; Group × Age: F=66.5, p<0.001; Group × Week: F=0.9, p=0.36; Group × Time: F=0.9, p=0.48; Age × Week: F=2.8, p=0.10; Age × Time: F=0.8, p=0.51; Week x Time: F=1.4, p=0.23; Group × Age × Week: F=10.6, p=0.001; Group × Age × Time: F=0.5, p=0.76; Group × Week × Time: F=0.2, p=0.94; Age × Week × Time: F=1.3, p=0.28; Group × Age × Week × Time: F=0.5, p=0.73.
B: Thermic effect of food in Black participants: Group: F=18.5, p<0.001; Age: F=107.9, p<0.001; Week: F=2.4, p=0.12; Time: F=183.1, p<0.001; Group × Age: F=6.6, p=0.01; Group × Week: F=4.4, p=0.04; Group × Time: F=0.6, p=0.64; Age × Week: F=1.9, p=0.17; Age × Time: F=4.1, p=0.003; 30: F=0.8, p=0.52; Group × Age × Week: F=0.1, p=0.778; Group × Age × Time: F=0.5, p=0.717; Group × Week × Time: F=0.3, p=0.91; Age × Week × Time: F=0.3, p=0.85; Group × Age × Week × Time: F=0.2, p=0.92.
C: Liver fat: Race: F=3.2, p=0.08; Group: F=10.1, p=0.003; Age: F=107.5, p<0.001; Week: F=3.1, p=0.09; Group × Race: F=0.6, p=0.43; Group × Age: F=0, p=0.93; Group × Week: F=17.1, p<0.001; Race × Age: F=6.7, p=0.02; Race × Week: F=1.9, p=0.18; Age × Week: F=0.8, p=0.39; Race × Group × Age: F=6.7, p=0.02; Race × Group × Week: F=2.6, p=0.12; Group × Age × Week: F=0.1, p=0.73; Race × Age × Week: F=0.2, p=0.70; Group × Race × Age × Week: F=0.8, p=0.34.
D: Intramyocellular lipids: Race: F=0.4, p=0.56; Group: F=0.7, p=0.415; Age: F=0.4, p=0.541; Week: F=0, p=0.988; Group × Race: F=0.8, p=0.386; Group × Age: F=14.1, p<0.001; Group × Week: F=5.8, p=0.023; Race × Age: F=0, p=0.917; Race × Week: F=0.3, p=0.605; Age × Week: F=3.7, p=0.066; Race × Group × Age: F=16.9, p<0.001; Race × Group × Week: F=0.1, p=0.722; Group × Age × Week: F=0.1, p=0.768; Race × Age × Week: F=0.2, p=0.677; Group × Race × Age × Week: F=0, p=0.935.

© 2020 Kahleova H et al. *JAMA Network Open.*

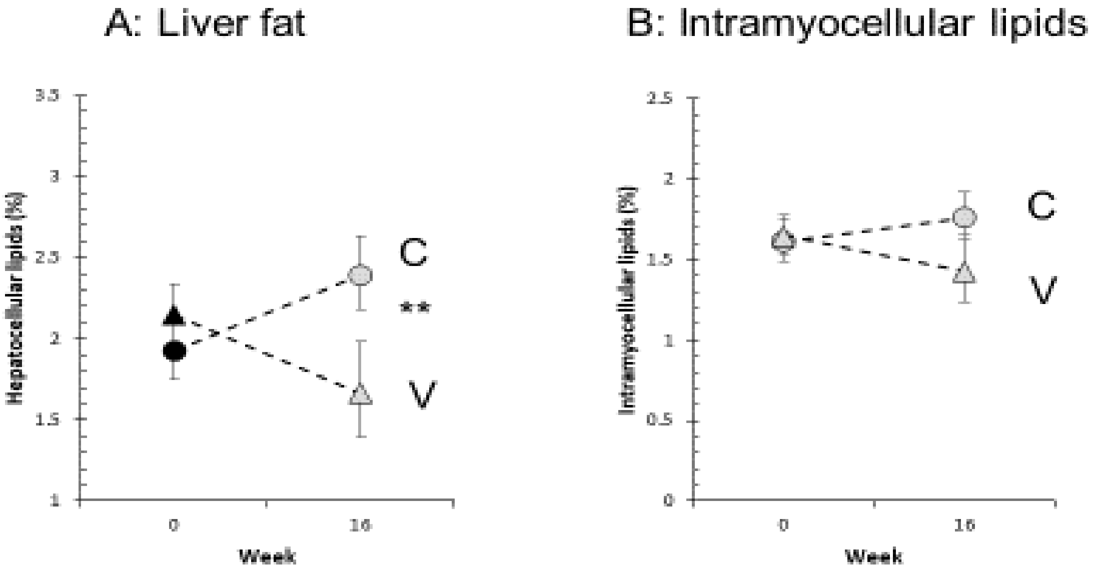

Supplemental Figure 2. Changes in liver fat (A), and intramyocellular lipids (B) after adjustment for baseline BMI.

Circles are used for the control group (C) and triangles for the vegan group (V).

Full symbols are used for week 0 and empty symbols for week 16.

A: Liver fat: Group: F=2.5, p=0.125; Baseline BMI: F=6.4, p=0.02; Week: F=0.1, p=0.82; Group × BMI: F=9.5, p=0.005; Group × Week: F=8.3, p=0.008; BMI × Week: F=0.8, p=0.39; Group × BMI × Week: F=0.1, p=0.71.

B: Intramyocellular lipids: Group: F=1.8, p=0.19; Baseline BMI: F=4.6, p=0.04; Week: F=0.2, p=0.70; Group × BMI: F=1.7, p=0.20; Group × Week: F=2.7, p=0.11; BMI × Week: F=0, p=0.94; Group × BMI × Week: F=0.1, p=0.79.

© 2020 Kahleova H et al. *JAMA Network Open.*

A: Energy intake and body weight

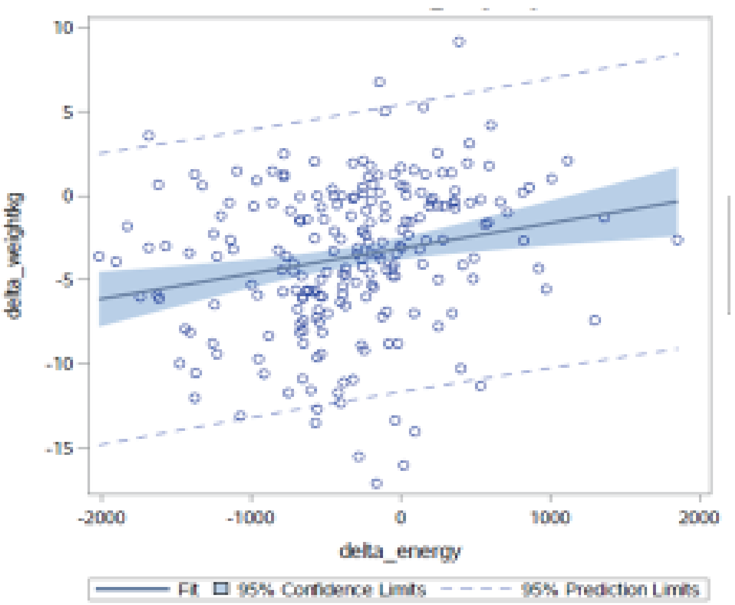

B: Postprandial energy expenditure and body weight

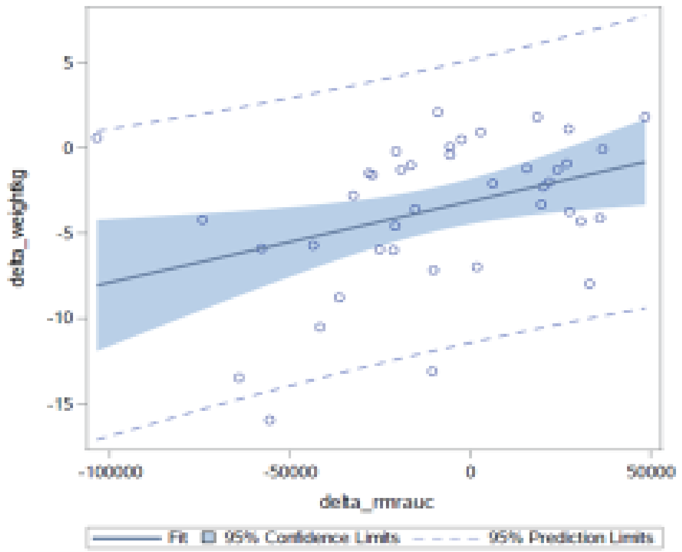

Supplemental Figure 3. Linear regression models for changes in energy intake and body weight (A) and postprandial energy expenditure (AUC, area under the curve) and body weight (B).
A: r=0.21; p=0.001; every change in energy intake of 100 kcal/day was associated with a change of body weight of 0.15 kg.
B: r=0.38; p=0.02; every change in postprandial energy expenditure of 10,000 units was associated with a change in body weight of 0.476 kg.

© 2020 Kahleova H et al. *JAMA Network Open.*
