# Supplementary material for: Effect of a Low-Fat Vegan Diet on Body Weight, Insulin Sensitivity, Postprandial Metabolism, and Intramyocellular and Hepatocellular Lipid Levels in Overweight Adults: A Randomized Clinical Trial
Source: JAMA Netw Open. 2020 Nov 30;3(11):e2025454. doi: 10.1001/jamanetworkopen.2020.25454 (PMC7705596; doi:10.1001/jamanetworkopen.2020.25454)
Supplement: Supplement 3. — Data Sharing Statement [file jamanetwopen-e2025454-s003.pdf]

Data Sharing Statement

Kahleova. Effect of a Low-Fat Vegan Diet on Body Weight, Insulin Sensitivity, Postprandial Metabolism, and Intramyocellular and Hepatocellular Lipid Levels in Overweight Adults. *JAMA Netw Open*. Published November 16, 2020. 10.1001/jamanetworkopen.2020.25454

Data
Data available: Yes
Data types: Deidentified participant data
How to access data: The data will be available upon request at hkahleova@pcrm.org
When available: With publication

Supporting Documents
Document types: None

Additional Information
Who can access the data: The data will be made available to researchers whose proposed use of the data has been approved.
Types of analyses: Meta-analyses, systematic reviews
Mechanisms of data availability: After approval of a proposal
Any additional restrictions: N/A
